# Bias and precision of methods for estimating the difference in restricted mean survival time from an individual patient data meta-analysis

**DOI:** 10.1186/s12874-016-0137-z

**Published:** 2016-03-29

**Authors:** Béranger Lueza, Federico Rotolo, Julia Bonastre, Jean-Pierre Pignon, Stefan Michiels

**Affiliations:** Gustave Roussy, Université Paris-Saclay, Service de biostatistique et d’épidémiologie, F-94805 Villejuif, France; Université Paris-Saclay, Univ. Paris-Sud, UVSQ, CESP, INSERM, F-94085 Villejuif, France; Ligue Nationale Contre le Cancer meta-analysis platform, Gustave Roussy, F-94085 Villejuif, France

**Keywords:** Restricted mean survival time, Survival benefit, Meta-analysis, Multicenter clinical trial, Survival analysis, Simulation study

## Abstract

**Background:**

The difference in restricted mean survival time ($rmstD\left({t}^{\ast}\right)$), the area between two survival curves up to time horizon ${t}^{\ast }$, is often used in cost-effectiveness analyses to estimate the treatment effect in randomized controlled trials. A challenge in individual patient data (IPD) meta-analyses is to account for the trial effect. We aimed at comparing different methods to estimate the $rmstD\left({t}^{\ast}\right)$ from an IPD meta-analysis.

**Methods:**

We compared four methods: the area between Kaplan-Meier curves (experimental vs. control arm) ignoring the trial effect (Naïve Kaplan-Meier); the area between Peto curves computed at quintiles of event times (Peto-quintile); the weighted average of the areas between either trial-specific Kaplan-Meier curves (Pooled Kaplan-Meier) or trial-specific exponential curves (Pooled Exponential). In a simulation study, we varied the between-trial heterogeneity for the baseline hazard and for the treatment effect (possibly correlated), the overall treatment effect, the time horizon ${t}^{\ast }$, the number of trials and of patients, the use of fixed or DerSimonian-Laird random effects model, and the proportionality of hazards. We compared the methods in terms of bias, empirical and average standard errors. We used IPD from the Meta-Analysis of Chemotherapy in Nasopharynx Carcinoma (MAC-NPC) and its updated version MAC-NPC2 for illustration that included respectively 1,975 and 5,028 patients in 11 and 23 comparisons.

**Results:**

The Naïve Kaplan-Meier method was unbiased, whereas the Pooled Exponential and, to a much lesser extent, the Pooled Kaplan-Meier methods showed a bias with non-proportional hazards. The Peto-quintile method underestimated the $rmstD\left({t}^{\ast}\right)$, except with non-proportional hazards at ${t}^{\ast }$= 5 years. In the presence of treatment effect heterogeneity, all methods except the Pooled Kaplan-Meier and the Pooled Exponential with DerSimonian-Laird random effects underestimated the standard error of the $rmstD\left({t}^{\ast}\right)$. Overall, the Pooled Kaplan-Meier method with DerSimonian-Laird random effects formed the best compromise in terms of bias and variance. The $rmstD\left({t}^{\ast },=,10,\kern0.5em ,\mathrm{years}\right)$ estimated with the Pooled Kaplan-Meier method was 0.49 years (95 % CI: [−0.06;1.03], *p* = 0.08) when comparing radiotherapy plus chemotherapy vs. radiotherapy alone in the MAC-NPC and 0.59 years (95 % CI: [0.34;0.84], *p* < 0.0001) in the MAC-NPC2.

**Conclusions:**

We recommend the Pooled Kaplan-Meier method with DerSimonian-Laird random effects to estimate the difference in restricted mean survival time from an individual-patient data meta-analysis.

**Electronic supplementary material:**

The online version of this article (doi:10.1186/s12874-016-0137-z) contains supplementary material, which is available to authorized users.

## Background

In cost-effectiveness analysis, a commonly used survival measure is the restricted mean survival time (RMST). It estimates the life expectancy for one treatment arm up to a certain time horizon ${t}^{\ast }$ [1–4]. The difference in restricted mean survival time ($rmstD\left({t}^{\ast}\right)$) can thus quantify the treatment effect expressed in terms of life years gained. The $rmstD\left({t}^{\ast}\right)$ is an appealing outcome measure as it is valid even in case of non-proportional hazards [5]. Moreover, as an absolute outcome, the interpretation of the $rmstD\left({t}^{\ast}\right)$ is considered more intuitive from the clinician point of view than the hazard ratio (HR) which is a relative measure of the treatment effect [3, 5, 6]. Recent studies have compared methods to estimate the RMST including extrapolation beyond the trial follow-up [7–10]. However, these studies focused on the use of a single randomized clinical trial and not specifically on multicenter clinical trials nor meta-analyses.

In meta-analyses or in multicenter clinical trials, there is a need to take into account the trial or center effect to avoid the Simpson’s paradox that may lead to biased estimates [11–13]. Different authors have discussed the use of Cox models with either stratification or fixed effect, or random effects to account for the center effect in a multicenter clinical trial [14–16] or the trial effect in a meta-analysis [17–20] in presence of baseline hazard heterogeneity and/or treatment effect heterogeneity between centers or trials. Several papers have also compared one-stage or two-stage methods to estimate the hazard ratio in individual patient data (IPD) meta-analyses [20–22]. All these studies focused on the estimation of the treatment effect through the use of the hazard ratio, but so far only one has focused on the use of the $rmstD\left({t}^{\ast}\right)$ in IPD meta-analyses [6]. In this latter study, Wei and colleagues investigated three two-stage methods to estimate the $rmstD\left({t}^{\ast}\right)$ from an IPD meta-analysis: two non-parametric methods – one based on pseudo-values [23] and one based on the Kaplan-Meier estimate – and a flexible parametric survival model [24]. In their study, the $rmstD\left({t}^{\ast}\right)$ was estimated as an aggregation of the $rmstD\left({t}^{\ast}\right)$ estimated in each trial using a fixed effect meta-analysis model. The authors showed via simulations and two case studies that the three methods produced similar results in terms of bias and coverage probability of the confidence intervals.

In the present paper, we aimed at extending the first study from Wei et al*.* [6] by comparing more methods for estimating the $rmstD\left({t}^{\ast}\right)$ from an IPD meta-analysis in a broader range of scenarios. We also designed a more realistic simulation study with random effects to induce between-trial heterogeneity, both in terms of baseline hazard and of treatment effect. We considered only one of the non-parametric methods studied by Wei and colleagues – the one pooling Kaplan-Meier estimates – as they found similar results for the three methods. We further considered a parametric method – pooling exponential estimates – and two other non-parametric methods: one naïve method that does not account for trial effect and an actuarial survival method developed by Peto, classically used in IPD meta-analyses for computing survival curves [25–27]. In simulations, we varied not only the treatment effect size and the time horizon ${t}^{\ast }$, as previously done by Wei and colleagues, but also the baseline hazard heterogeneity, the treatment effect heterogeneity, the correlation between these two random effects, the number of trials and the number of patients per trial, the use of fixed effect and DerSimonian-Laird random effects model [28], and departure from the assumption of proportional hazards.

In the ‘Methods’ section we describe the $rmstD\left({t}^{\ast}\right)$ and the different survival analysis methods for estimating the $rmstD\left({t}^{\ast}\right)$ that we investigate in this paper. Section ‘Simulation study’ describes the design of the simulation study, how to estimate the true $rmstD\left({t}^{\ast}\right)$, the simulation scenarios and the evaluation criteria, and presents the simulation results. Section ‘Application’ provides two examples using IPD meta-analyses in nasopharynx carcinoma. We end with a discussion and our conclusion regarding the choice of a particular method to estimate the $rmstD\left({t}^{\ast}\right)$ from an IPD meta-analysis. Of note, the investigated methods can also be used for the estimation of the $rmstD\left({t}^{\ast}\right)$ in multicenter clinical trials.

## Methods

### Difference in restricted mean survival time

Let *T* be the survival time random variable with distribution *F(t)*. The mean survival time restricted at a specified time ${t}^{\ast }$ is defined as

1 $$ RMST\left({t}^{\ast}\right)={\displaystyle {\int}_0^{t^{\ast }}S(t)dt,} $$

where *S(t)* = 1 – *F(t)* is the survival function. The *RMST*${t}^{\ast }$ corresponds graphically to the area under the survival curve *S(t)* up to ${t}^{\ast }$. The difference in restricted mean survival time ($rmstD\left({t}^{\ast}\right)$) between the experimental arm and the control arm (noted 1 and 0) is defined as

2 $$ rmstD\left({t}^{\ast}\right)=RMS{T}_1\left({t}^{\ast}\right)-RMS{T}_0\left({t}^{\ast}\right) $$

The variance $\widehat{Var}\left( rmstD\left({t}^{\ast}\right)\right)$ can be estimated as [29]:

3 $$ \widehat{Var}\left( rmstD\left({t}^{\ast}\right)\right)=\widehat{Var}\left(RMS{T}_1\left({t}^{\ast}\right)\right)+\widehat{Var}\left(RMS{T}_0\left({t}^{\ast}\right)\right) $$

As opposed to the relative hazard ratio, the $rmstD\left({t}^{\ast}\right)$ is an absolute outcome which depends both on the baseline hazard and on the relative treatment effect.

Wei et al. [6] also proposed the use of the relative difference in restricted mean survival time defined as

4 $$ rmstRD\left({t}^{\ast}\right)= rmstD\left({t}^{\ast}\right)/{t}^{\ast } $$

The $rmstD\left({t}^{\ast}\right)$ varies between 0 and 1 and can be interpreted as a percentage. Its variance can be estimated as $\widehat{Var}\left[ rmstD\left({t}^{\ast}\right)\right]/{\left({t}^{\ast}\right)}^2$.

### Methods for estimating the difference in restricted mean survival time

We investigated four methods for estimating the difference in restricted mean survival time $rmstD\left({t}^{\ast}\right)$ from an IPD meta-analysis: 1) the Naïve Kaplan-Meier, which pools the data, ignoring the trial effect, 2-3) the Pooled Kaplan-Meier and Pooled Exponential methods, which use a two-stage approach to combine ${rmstD}_j\left({t}^{\ast}\right)$ estimated in each trial *j*, and 4) the Peto-quintile method, which uses survival functions derived from a pooled hazard ratio estimated with a two-stage approach in order to take into account the trial effect.

#### Naïve Kaplan-Meier

The most basic method to estimate the $rmstD\left({t}^{\ast}\right)$ is to consider the IPD meta-analysis as a single large trial. Under this approach, the $rmstD\left({t}^{\ast}\right)$ is estimated as the area between the Kaplan-Meier curves of the experimental and the control arm, obtained by pooling the data from all the trials, thus ignoring the trial effect [30]:

5 $$ \widehat{rmst}D\left({t}^{\ast}\right)={\displaystyle {\sum}_{i=1}^{D_1-1}{\widehat{S}}_1\left({t}_{1,i}\right)}\left({t}_{1,i+1}-{t}_{1,i}\right)-{{\displaystyle {\sum}_{i=1}^{D_0-1}\widehat{S}}}_0\left({t}_{0,i}\right)\left({t}_{0,i+1}-{t}_{0,i}\right) $$

with *Ŝ*_*arm*_(*t*_*arm*,0_) = 0, *t*_*arm*,0_ = 0, *D*_*arm*_ the number of distinct event times (*t*_*arm,1*_ < *t*_*arm,2*_ < … < *t*_*arm,D*_) and *Ŝ*_*arm*_(*t*) the Kaplan-Meier estimator for the experimental arm and the control arm (noted 1 and 0). The variance of the $rmstD\left({t}^{\ast}\right)$ was estimated analytically by the delta method for the Naïve Kaplan-Meier (details are provided in Additional file 1).

#### Pooled Kaplan-Meier and Pooled Exponential

In order to take into account the trial effect, a different strategy consists in estimating the ${rmstD}_j\left({t}^{\ast}\right)$ within each trial *j* and then to combine the trial-specific results into a pooled estimate. In the Pooled Kaplan-Meier and Pooled Exponential methods, which are both two-stage approaches, we first estimated the ${rmstD}_j\left({t}^{\ast}\right)$ in each trial *j* as the area between either trial-specific Kaplan-Meier curves

6 $$ \widehat{rmst}{D}_j\left({t}^{\ast}\right)={\displaystyle {\sum}_{i=1}^{D_{j,1}-1}{\widehat{S}}_{j,1}\left({t}_{j,1,i}\right)\left({t}_{j,1,i+1}-{t}_{j,1,i}\right)-{\displaystyle {\sum}_{i=1}^{D_{j,0}-1}{\widehat{S}}_{j,0}\left({t}_{j,0,i}\right)\left({t}_{j,0,i+1}-{t}_{j,0,i}\right)}} $$

or trial-specific exponential curves

7 $$ \widehat{rmst}{D}_j\left({t}^{\ast}\right)={\displaystyle {\int}_0^{t^{\ast }}{e}^{-{\widehat{\lambda}}_{j,1}t}dt-{\displaystyle {\int}_0^{t^{\ast }}{e}^{-{\widehat{\lambda}}_{j,0}t}dt=\frac{1-{e}^{-{\widehat{\lambda}}_{j,1}{t}^{\ast }}}{{\widehat{\lambda}}_{j,1}}}}-\frac{1-{e}^{-{\widehat{\lambda}}_{j,0}{t}^{\ast }}}{{\widehat{\lambda}}_{j,0}} $$

with for each trial *j: Ŝ*_*j*,*arm*_(*t*_*j*,*arm*,0_) = 0, *t*_*j*,*arm*,0_ = 0, *D*_*j*,*arm*_ the number of distinct event times (*t*_*j,arm,1*_ < *t*_*j,arm,2*_ < … < *t*_*j,arm,D*_), *Ŝ*_*j*,*arm*_(*t*) the Kaplan-Meier estimator, and ${\widehat{\lambda}}_{j, arm}$ the maximum likelihood estimate of the scale parameter for the exponential distribution for the experimental arm and the control arm (noted 1 and 0).

Then, we combined the ${rmstD}_j\left({t}^{\ast}\right)$ by using a fixed effect or a DerSimonian-Laird random effects [28] meta-analysis model (see Additional file 1). The variance of each ${rmstD}_j\left({t}^{\ast}\right)$ was estimated analytically by the delta method for the Pooled Kaplan-Meier and Pooled Exponential methods (details are provided in Additional file 1).

#### Peto-quintile

The actuarial method was developed by Peto to compute the survival curves taking into account the trial effect in IPD meta-analyses [25–27, 31]. In this case, the survival probabilities in the two arms are estimated at the end of predetermined time intervals *i* based on the estimated survival probability *p*_*i*_ in the overall population and a pooled hazard ratio *HR*_*i,pooled*_. The survival probability for the overall population is estimated as

8 $$ \widehat{p_{\iota }}= \exp \left(-\frac{D_i}{P{I}_i}\right) $$

where *D*_*i*_ is the number of deaths during interval *i* and *PI*_*i*_ the total number of person-intervals in the *i*-th interval. One person-interval is equivalent to one person-year when the time interval chosen is 1 year. The pooled hazard ratio in the interval, ${\widehat{HR}}_{i, pooled},$ is estimated using a fixed effect meta-analysis model to aggregate the different ${\widehat{HR}}_{i,j}$ estimated in each trial *j*. The survival probabilities at the end of each interval *i* in the control arm (*p*_*0,i*_) and in the experimental arm (*p*_*1,i*_) are estimated as follows:

9 $$ \widehat{p_{0,\iota }}=\widehat{p_{\iota }}-\left[0.5\widehat{p_{\iota }}\left(\widehat{p_{\iota }}-1\right) log\left({\widehat{HR}}_{i, pooled}\right)\right] $$

10 $$ \widehat{p_{1,\iota }}=\widehat{p_{\iota }}+\left[0.5\widehat{p_{\iota }}\left(\widehat{p_{\iota }}-1\right) log\left({\widehat{HR}}_{i, pooled}\right)\right] $$

The survival probability at time *t* in each arm is the product of the survival probabilities across *n*_i_ intervals up to *t*

11 $$ {\widehat{S}}_{peto,0}(t)={\displaystyle {\prod}_{i=1}^{n_i}{p}_{0,i}}\kern0.5em \mathrm{and}\kern0.5em {\widehat{S}}_{peto,1}(t)={\displaystyle {\prod}_{i=1}^{n_i}{p}_{1,i}} $$

In the present work, we extended this method by also pooling the ${\widehat{HR}}_{i,j}$ using a DerSimonian-Laird random effects meta-analysis model (see Additional file 1). Furthermore, we used time intervals *i* based on the quintiles of the overall number of deaths occurring before ${t}^{\ast }$ in the meta-analysis and therefore we called this method the Peto-quintile method. The $rmstD\left({t}^{\ast}\right)$ was then estimated as the area between the two survival curves defined by *Ŝ*_*Peto*,0_(*t*) and *Ŝ*_*Peto*,1_(*t*).

12 $$ \widehat{rmstD}\left({t}^{\ast}\right)={\displaystyle {\sum}_{i=0}^4\frac{\left({t}_{i+1}-{t}_i\right)}{2}\left[\left({\widehat{S}}_{Peto,1}\left({t}_{i+1}\right)-{\widehat{S}}_{Peto,0}\left({t}_{i+1}\right)\right)+\left({\widehat{S}}_{Peto,1}\left({t}_i\right)-{\widehat{S}}_{Peto,0}\left({t}_i\right)\right)\right]} $$

where *t*_*0*_ = 0 and $\left({t}_1,..,\kern0.2em {t}_5={t}^{*}\right)$ denotes the time intervals based on the quintiles of events.

A 50-replicate non-parametric bootstrap was used to estimate the variance of the $rmstD\left({t}^{\ast}\right)$ for the Peto-quintile method.

#### Follow-up differences across trials and extrapolation

We used the extrapolation method proposed by Brown et al*.* [32] for Naïve Kaplan-Meier and Pooled Kaplan-Meier, to extrapolate the survival function beyond the last observed event time (*t*_*max*_) until ${t}^{\ast }$, if needed (e.g. in case of potential follow-up difference across trials) [10, 32]. The Brown extrapolation method consists in completing the tail of the Kaplan-Meier survival curve by an exponential curve. The estimated survival function for *t* > *t*_*max*_ is:

13 $$ {\widehat{S}}_{Brown}(t)= \exp \left\{t\times log\left[\widehat{S}\left({t}_{max}\right)\right]/{t}_{max}\right\} $$

where *Ŝ* (*t*) is the Kaplan-Meier estimator of the survival function.

No extrapolation was needed for Pooled Exponential as a parametric model is used. Concerning the Peto-quintile method, at least one event is needed overall in the meta-analysis in each arm and at each time interval *i* for survival probabilities *p*_*0,i*_ and *p*_*1,i*_ to be computed. This was always the case, even in case of a potential difference in follow-up across trials, as each time interval contained by definition one fifth of the deaths occurring before ${t}^{\ast }$. It is worth noting that if ${t}^{\ast }$ is greater than *t*_*max*_ the last observed event in the whole meta-analysis, the method estimates the event rate *p*_*i*_ and the pooled hazard ratio *HR*_*i,pooled*_ until *t*_*max*_ and implicitly extrapolates the survival in the remaining interval $\left[{t}_{max},{t}^{*}\right]$.

## Simulation study

### Design of the simulation study

We simulated survival times of *N* patients from *J* trials, each of size *n*_*j*_ with ∑_*j* = 1_^*J*^*n*_*j*_ = *N*. We defined the hazard function for a trial *j* ϵ {1 , …, *J*} as

14 $$ \lambda \left(t;x\Big|{A}_j={a}_j,{B}_j={b}_j\right)={\lambda}_0(t) exp\left\{{a}_j+\left(\beta +{b}_j\right)x\right\} $$

where *λ*_*0*_*(t)* is the baseline hazard function, *A*_*j*_ is a trial-specific random quantity affecting the baseline hazard with Var(*A*_*j*_) = *σ*^*2*^, and *B*_*j*_ is a trial-specific random quantity affecting the treatment effect with Var(*B*_*j*_) = *τ*^*2*^, *β* is the overall treatment effect, and *χ* is the binary treatment variable, coded +1/2 for experimental arm, −1/2 for control in order to obtain equal heterogeneity in both arms [19, 21, 22]. Of note, the covariance between the two random effects is defined by cov(*A*_*j*_,*B*_*j*_) = *ρστ* with *ρ* the correlation between *A*_*j*_ and *B*_*j*_.

We used an exponential distribution for the baseline hazard function λ_0_(*t*) = (log(2)/5)*t*, corresponding to a median survival time of 5 years. Independent and non-informative right-censoring was induced by setting the recruitment time at 3 years for all trials and varying the maximum follow-up time uniformly between 2 and 9 years across trials to replicate the typical difference in observed follow-up between trials included an IPD meta-analysis.

We induced between-trial heterogeneity by generating random values a_j_ and b_j_ from binomial distributions for the baseline hazard and the treatment effect. The use of a discrete distribution allowed us to derive straightforwardly the true difference in restricted mean survival time ($rmstD\left({t}^{\ast}\right)$). The binomial random variables were centered and properly rescaled in order to obtain the desired variances *σ*^*2*^ and *τ*^*2*^:

15 $$ A\sim \left[ Bin\left(n=50,p=0.5\right)-25\right]\cdot \sigma /\sqrt{12.5} $$

16 $$ B\sim \left[ Bin\left(n=50,p=0.5\right)-25\right]\cdot \tau /\sqrt{12.5} $$

The rationale for the arbitrary choice of *n* = 50 was that the distribution approximated well a continuous distribution, while allowing easy computation of the true $rmstD\left({t}^{\ast}\right)$.

### True difference in restricted mean survival time

Based on our simulation model, the difference in restricted mean survival time is defined as

17 $$ rmstD\left({t}^{*}\right)={\displaystyle {\int}_0^{t^{*}}S\left(t;x=\frac{1}{2}\right)dt-}{\displaystyle {\int}_0^{t^{*}}S\left(t;x=-\frac{1}{2}\right)(t)dt} $$

18 $$ rmstD\left({t}^{*}\right)={\displaystyle {\int}_0^{t^{*}}\left[{\displaystyle {\int}_{\mathcal{K}}S}\left(t;x=\frac{1}{2}\Big|{a}_j,{b}_j\right)d{F}_{A,B}\left(a,b\right)\right]dt-{\displaystyle {\int}_0^{t^{*}}\left[{\displaystyle {\int}_{\mathcal{K}}S}\left(t;x=-\frac{1}{2}\Big|{a}_j,{b}_j\right)d{F}_{A,B}\left(a,b\right)\right]dt}} $$

where $\mathcal{K}={\left\{\left({a}_k,{b}_k\right)\right\}}_{k=1,\dots,\ K}$ is the support of the bivariate variable (A,B). The joint distribution *F*_*A,B*_*(a,b)* is defined by the probabilities *p*_*k*_ = ℙ(*A* = *a*_*k*_, *B* = *b*_*k*_ )_*k* = 1,…, *K*_ of all the *K* admissible couples of values (*a*_*k*_,*b*_*k*_). Thanks to the use of a discrete joint probability distribution *F*_*A,B*_*(a,b)*, the integral in equation (18) boils down to a sum over the *K* points belonging to its support $\mathcal{K}$:

19 $$ rmstD\left({t}^{\ast}\right)={\displaystyle {\sum}_{k=1}^K{p}_k{rmstD}_k\left({t}^{\ast}\right)}, $$

with the conditional restricted mean survival time ${rmstD}_k\left({t}^{\ast}\right)$ defined for a couple (*a*_*k*,_*b*_*k*_) as:

20 $$ rmst{D}_k\left({t}^{*}\right)={\displaystyle {\int}_0^{t^{*}} \exp \left(-\frac{ \log (2)}{5}\cdot t\cdot {e}^{a_k+1/2\left(\beta +{b}_k\right)}\ \right)dt-{\displaystyle {\int}_0^{t^{*}} \exp \left(-\frac{ \log (2)}{5}\cdot t\cdot {e}^{a_k-1/2\left(\beta +{b}_k\right)}\ \right)dt}} $$

### Simulation scenarios

In different scenarios we varied the strength of between-trial heterogeneity for the baseline hazard (low with *σ*^*2*^ = 0.01 and high with *σ*^*2*^ = 0.10) and for the treatment effect (*τ*^*2*^ = 0.01, 0.10). We performed the main analysis with uncorrelated random effects (*ρ* = 0), however, in a sensitivity analysis, we studied the impact of a negative correlation between *A*_*j*_ and *B*_*j*_ (*ρ* = −0.8). We considered different values for the number of trials and patients per trial: (*J* = 5, *n*_*j*_ = 200) and (*J* = 20, *n*_*j*_ = 100) and for the size of the overall treatment effect (*β* = 0, ±0.2, ±0.7). We also studied the impact of the time horizon of restriction at ${t}^{\ast }$= 5 years and ${t}^{\ast }$= 10 years. These two values were chosen to illustrate a scenario in which all trials still have patients at risk at ${t}^{\ast }$ (5 years) and a scenario in which some trials’ follow-up are shorter than the time of restriction (${t}^{\ast }$= 10 years). The average administrative censoring rate ranged across scenarios from 49 to 52 % at ${t}^{\ast }$= 5 years and from 38 to 40 % at ${t}^{\ast }$= 10 years. In the case of no overall treatment effect at all (*β* = 0, *τ*^*2*^ = 0) and no baseline heterogeneity (*σ*^*2*^ = 0), the restricted mean survival time was equal to 3.6 years at ${t}^{\ast }$= 5 years and 5.4 years at ${t}^{\ast }$= 10 years in both arms. The influence of non-proportional hazards was examined using a piecewise exponential distribution with a deleterious treatment effect (*β’* = −*β*, with *β* ≤ 0) in the first 2 years and a beneficial treatment effect (*β*) afterwards.

### Evaluation criteria

We simulated 1,000 meta-analyses for each scenario and compared the four methods using: the average bias, defined as the average of the estimated $rmstD\left({t}^{\ast}\right)$ minus the true value; the empirical standard error (ESE), defined as the standard deviation of the $rmstD\left({t}^{\ast}\right)$ over the replicates; and the average standard error (ASE), defined as the average of the estimated standard errors [33].

With the exception of the Naïve Kaplan-Meier method, the methods were not available in standard statistical software. We implemented the methods and performed the simulation study using R version 3.1.3 (R Foundation, Vienna, Austria). The R code is available from the authors upon request.

## Results

**Fig. 1:**
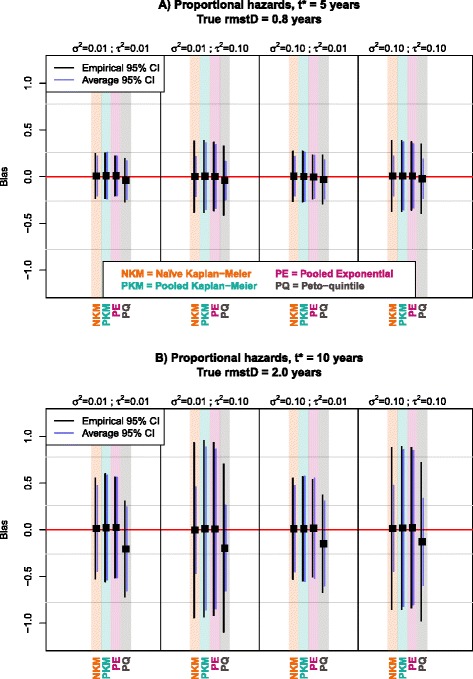
Graphical comparison at 5 years (panel **a**) and at 10 years (panel **b**) of methods in terms of bias, empirical and average standard error with proportional hazards; 5 trials and 200 patients per trial; *β* = −0.7. Black squares represent the average bias of the rmstD estimated by each method for a particular scenario. Black and purple vertical lines represent the 95 % confidence intervals of the bias based on respectively the empirical and average standard errors. The horizontal red line indicates the absence of bias in the rmstD estimation. *β*: Size of treatment effect (=log(HR)); σ^2^: baseline hazard heterogeneity; τ^2^: treatment effect heterogeneity; CI: Confidence interval; rmstD: difference in restricted mean survival time

For all scenarios, there was almost no bias in the case of no treatment effect (*β* = 0). When there was a beneficial treatment effect (*β* = −0.2 or −0.7), Peto-quintile underestimated the $rmstD\left({t}^{\ast}\right)$, notably on the long term (${t}^{\ast }$= 10 years). The Pooled Exponential and, to a much lesser extent, the Pooled Kaplan-Meier methods showed a bias in the case of non-proportional hazards. In all these cases, whenever a method showed a bias, the bias increased with |*β*| (Table 1 and Fig. 1).

**Table 1:** Simulation results for comparisons of methods in estimating the difference in restricted mean survival time. Scenario with 5 trials and 200 patients per trial and with proportional hazards

Heterogeneity scenario	Methods	*β* = 0						*β* = −0.7					
		*t* ^*^ = 5 True *rmstD* = 0			*t* ^*^ = 10 True *rmstD* = 0			*t* ^*^ = 5 True *rmstD* = 0.8			*t* ^*^ = 10 True *rmstD* = 2.0		
		Bias	ESE	ASE	Bias	ESE	ASE	Bias	ESE	ASE	Bias	ESE	ASE
(σ^2^,τ^2^) = (0.01;0.01)	Naïve Kaplan-Meier	0.00	0.12	0.11	0.00	0.28	0.24	0.01	0.12	0.11	0.01	0.28	0.23
	Pooled Kaplan-Meier	0.00	0.12	0.13	0.00	0.29	0.30	0.01	0.12	0.13	0.02	0.30	0.29
	Pooled Exponential	0.00	0.10	0.11	0.00	0.27	0.28	0.01	0.11	0.11	0.02	0.27	0.27
	Peto-quintile	0.00	0.11	0.10	0.00	0.24	0.21	−0.04	0.12	0.10	−0.21	0.26	0.23
(σ^2^,τ^2^) = (0.01;0.10)	Naïve Kaplan-Meier	0.00	0.19	0.11	0.01	0.49	0.24	0.00	0.19	0.11	0.00	0.48	0.23
	Pooled Kaplan-Meier	0.00	0.20	0.18	0.01	0.50	0.47	0.00	0.20	0.18	0.01	0.48	0.44
	Pooled Exponential	0.00	0.19	0.17	0.01	0.48	0.45	0.00	0.19	0.17	0.01	0.47	0.44
	Peto-quintile	0.00	0.18	0.10	0.00	0.43	0.22	−0.04	0.19	0.10	−0.20	0.46	0.23
(σ^2^,τ^2^) = (0.10;0.01)	Naïve Kaplan-Meier	−0.01	0.12	0.11	−0.01	0.28	0.24	0.00	0.14	0.11	0.01	0.28	0.24
	Pooled Kaplan-Meier	−0.01	0.12	0.13	0.00	0.28	0.29	0.00	0.14	0.13	0.01	0.28	0.29
	Pooled Exponential	0.00	0.10	0.11	−0.01	0.26	0.28	0.00	0.12	0.12	0.02	0.27	0.27
	Peto-quintile	−0.01	0.11	0.10	−0.01	0.23	0.22	−0.03	0.13	0.11	−0.15	0.26	0.23
(σ^2^,τ^2^) = (0.10;0.10)	Naïve Kaplan-Meier	0.00	0.18	0.11	0.00	0.45	0.24	0.01	0.19	0.11	0.01	0.44	0.24
	Pooled Kaplan-Meier	0.00	0.18	0.18	0.00	0.45	0.45	0.01	0.19	0.18	0.02	0.44	0.43
	Pooled Exponential	0.00	0.17	0.17	0.00	0.44	0.44	0.01	0.19	0.18	0.02	0.44	0.42
	Peto-quintile	0.00	0.17	0.10	0.00	0.40	0.22	−0.02	0.19	0.11	−0.13	0.43	0.24

A DerSimonian-Laird random effects meta-analysis model was used for Pooled Kaplan-Meier, Pooled Exponential and Peto-quintile

*β* Size of treatment effect (=log(HR)), *σ* ^*2*^ baseline hazard heterogeneity, *τ* ^*2*^ treatment effect heterogeneity, *ASE* average standard error, *CI* Confidence interval, *ESE* empirical standard error, *rmstD* difference in restricted mean survival time, *t* ^*^ time horizon

**Fig. 2:**
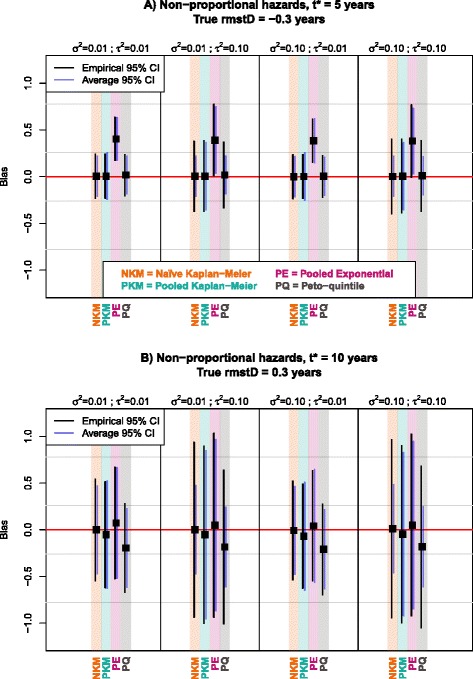
Graphical comparison at 5 years (panel **a**) and at 10 years (panel **b**) of methods in terms of bias, empirical and average standard error with non-proportional hazards; 5 trials and 200 patients per trial; *β* = −0.7. Black squares represent the average bias of the rmstD estimated by each method for a particular scenario. Black and purple vertical lines represent the 95 % confidence intervals of the bias based on respectively the empirical and average standard errors. The horizontal red line indicates the absence of bias in the rmstD estimation. *β*: Size of treatment effect (=log(HR)); σ^2^: baseline hazard heterogeneity; τ^2^: treatment effect heterogeneity; CI: Confidence interval; rmstD: difference in restricted mean survival time

In scenarios with higher treatment effect heterogeneity (*τ*^*2*^ = 0.10), all the methods had higher empirical standard error (ESE), as shown in the Figs. 1 and 2, and Tables 1 and 2. The standard error was estimated correctly (ASE = ESE) only with Pooled Kaplan-Meier and Pooled Exponential. It was generally underestimated (ASE < ESE) with the two other methods: the ASE was two-fold smaller than the ESE for the Naïve Kaplan-Meier and the Peto-quintile methods with *τ*^*2*^ = 0.10. When varying the baseline hazard heterogeneity between trials, no relevant impact was noted neither on the bias nor on the standard error.

**Table 2:** Simulation results for comparisons of methods in estimating the difference in restricted mean survival time. Scenario with 5 trials and 200 patients per trial, and with non-proportional hazards

Heterogeneity scenario	Methods	*β* = 0						*β* = −0.7					
		*t* ^*^ = 5 True *rmstD* = 0			*t* ^*^ = 10 True *rmstD* = 0			*t* ^*^ = 5 True *rmstD* = −0.3			*t* ^*^ = 10 True *rmstD* = 0.3		
		Bias	ESE	ASE	Bias	ESE	ASE	Bias	ESE	ASE	Bias	ESE	ASE
(σ^2^,τ^2^) = (0.01;0.01)	Naïve Kaplan-Meier	0.00	0.12	0.11	0.00	0.28	0.24	0.00	0.12	0.11	0.00	0.28	0.24
	Pooled Kaplan-Meier	0.00	0.12	0.13	0.01	0.29	0.30	0.01	0.12	0.13	−0.05	0.29	0.29
	Pooled Exponential	0.00	0.10	0.11	0.00	0.27	0.28	0.40	0.12	0.12	0.07	0.30	0.30
	Peto-quintile	0.00	0.11	0.10	0.00	0.24	0.21	0.02	0.11	0.10	−0.19	0.24	0.21
(σ^2^,τ^2^) = (0.01;0.10)	Naïve Kaplan-Meier	0.00	0.19	0.11	0.02	0.50	0.24	0.00	0.19	0.11	0.00	0.48	0.24
	Pooled Kaplan-Meier	0.01	0.19	0.18	0.02	0.50	0.46	0.00	0.19	0.18	−0.05	0.48	0.46
	Pooled Exponential	0.00	0.19	0.17	0.01	0.49	0.45	0.39	0.20	0.18	0.05	0.50	0.47
	Peto-quintile	0.00	0.18	0.10	0.01	0.43	0.22	0.02	0.18	0.10	−0.18	0.42	0.22
(σ^2^,τ^2^) = (0.10;0.01)	Naïve Kaplan-Meier	0.00	0.12	0.11	0.00	0.28	0.24	0.00	0.12	0.11	−0.01	0.27	0.24
	Pooled Kaplan-Meier	0.00	0.12	0.13	0.00	0.29	0.29	0.00	0.12	0.13	−0.07	0.28	0.29
	Pooled Exponential	0.00	0.10	0.11	0.00	0.27	0.28	0.38	0.12	0.12	0.04	0.30	0.31
	Peto-quintile	0.00	0.11	0.10	0.00	0.24	0.21	0.00	0.11	0.10	−0.21	0.25	0.22
(σ^2^,τ^2^) = (0.10;0.10)	Naïve Kaplan-Meier	−0.01	0.19	0.11	−0.01	0.46	0.24	0.00	0.20	0.11	0.01	0.49	0.24
	Pooled Kaplan-Meier	−0.01	0.19	0.18	−0.02	0.47	0.45	0.00	0.20	0.18	−0.05	0.48	0.45
	Pooled Exponential	−0.01	0.18	0.17	−0.01	0.45	0.44	0.38	0.20	0.18	0.05	0.50	0.46
	Peto-quintile	−0.01	0.18	0.10	−0.01	0.41	0.22	0.01	0.19	0.10	−0.18	0.44	0.22

A DerSimonian-Laird random effects meta-analysis model was used for Pooled Kaplan-Meier, Pooled Exponential and Peto-quintile

*β* Size of treatment effect (=log(HR)), *σ* ^*2*^ baseline hazard heterogeneity, *τ* ^*2*^ treatment effect heterogeneity, *ASE* average standard error, *CI* Confidence interval, *ESE* empirical standard error, *rmstD* difference in restricted mean survival time, *t* ^*^ time horizon

With both proportional and non-proportional hazards, for *β* = −0.7, the Peto-quintile method showed a bias which was negligible at ${t}^{\ast }$= 5 years but much higher at ${t}^{\ast }$= 10 years (up to 0.21 years; Fig. 1-b and Fig. 2-b). In the case of non-proportional hazards, which were incorporated using a piecewise exponential distribution with a deleterious treatment effect in the first 2 years and a beneficial treatment effect afterwards, Pooled Exponential was heavily biased at 5 years, with a bias of almost 0.40 years as compared to a true $rmstD\left({t}^{\ast },=,5\right)$ = −0.30 years (Fig. 2-a and Table 2). This bias suggests that the Pooled Exponential method failed to reflect the piecewise exponential distribution with *β’* = −*β* (*β* ≤ 0) for *t* ϵ [0;2] years and *β* for *t* > 2 years. However, this bias disappeared at 10 years, arguably because the true hazards were proportional between 2 and 10 years in our simulation set-up (Fig. 2-b) and the different effect in the first 2 years was thus attenuated. A small bias also arose for Pooled Kaplan-Meier at 10 years when the hazards were not proportional: a bias of around 0.05 years as compared to a true $rmstD\left({t}^{\ast },=,10\right)$ = 0.30 years (Fig. 2-b).

In terms of standard error, lower values were found for both ESE and ASE at ${t}^{\ast }$ = 5 years (Fig. 1-a and Fig. 2-a) as compared at ${t}^{\ast }$= 10 years (Fig. 1-b and Fig. 2-b), no matter the hazards were proportional or not.

The number of trials and the size of trials had no major impact in terms of bias. In meta-analyses of *J* = 20 trials and *n*_*j*_ = 100 patients per trial, all the methods had lower empirical and average standard errors than in meta-analyses of *J* = 5 trials and *n*_*j*_ = 200 patients per trial (see Additional file 1: Tables S1, 2, 3 and 4).

We also considered a deleterious treatment effect (*β* = +0.2, +0.7) but, as expected, results were not affected: the biased methods had biases that were reversed, and ASE and ESE remained unchanged (Additional file 1: Table S5).

The introduction of a negative correlation between *A*_*j*_ and *B*_*j*_ also had no major impact in terms of bias and standard error estimation, with the exception of the scenario with high baseline hazard and treatment variances (*σ*^*2*^ = *τ*^*2*^ = 0.10) for which ASE and ESE of all the methods were higher than with no correlation, notably for *β* = −0.7 (Additional file 1: Table S6).

When using a fixed effect meta-analysis model (Additional file 1: Table S7), for scenarios with high treatment effect heterogeneity (*τ*^*2*^ = 0.10), the Pooled Kaplan-Meier, Pooled Exponential and Peto-quintile methods exhibited a larger bias as compared with a DerSimonian-Laird random effects models used in Table 1. Furthermore, using a fixed effect model underestimated the standard error in general (ASE < <ESE).

## Application

We illustrate the four methods for estimating the $rmstD\left({t}^{\ast}\right)$ using IPD from the Meta-Analysis of Chemotherapy in Nasopharynx Carcinoma (MAC-NPC) Collaborative Group [34] and its updated version MAC-NPC2 [35] as these two IPD meta-analyses differed in terms of evidence of treatment effect heterogeneity. These IPD meta-analyses studied the addition of chemotherapy (CT) to radiotherapy (RT) in patients with nasopharynx carcinoma. For the estimation of the $rmstD\left({t}^{\ast}\right)$, we selected ${t}^{\ast }$= 5 years and ${t}^{\ast }$= 10 years, as these were the two time points of clinical interest in the publications of MAC-NPC and MAC-NPC2.

### Meta-Analysis of Chemotherapy in Nasopharynx Carcinoma (MAC-NPC)

The data from the MAC-NPC [34] included 1,975 patients in 11 treatment comparisons. The pooled HR estimated with a fixed effect model was 0.82 (95 % CI: [0.71;0.94]), indicating a significant improvement in overall survival with RT plus CT (*p* = 0.006). The treatment effect heterogeneity was significant (Q-test: *p* = 0.03; Higgins’ I^2^ = 50 %) which was explained by the timing of CT. The pooled HR estimated with a DerSimonian-Laird random effects model [28] was 0.82 (95 % CI: [0.66;1.02], *p* = 0.08).

**Fig. 3:**
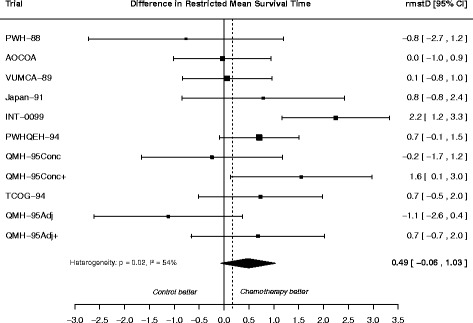
Forest plot for differences in restricted mean survival time estimated at 10 years using the Pooled Kaplan-Meier method with random effects applied to the MAC-NPC meta-analysis. Each trial is represented by a square, the center of which denotes the difference in restricted mean survival time (rmstD) for that trial comparison, with the horizontal lines showing the 95 % confidence intervals (CI). The size of the square is directly proportional to the amount of information contributed by the trial. The diamond represents the overall rmstD, with the center denoting the rmstD and the extremities the 95 % CI. The rmstDs are expressed in year

**Fig. 4:**
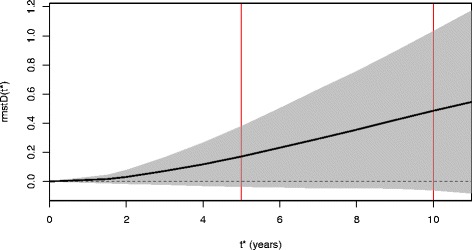
Difference in restricted mean survival time estimated using the Pooled Kaplan-Meier method with random effects as a function of the time horizon *t* ^*^ in the MAC-NPC meta-analysis. The solid black line represents the *rmstD*(*t* ^*^) plotted as a function of the horizon *t* ^*^. The dashed horizontal line indicates the absence of a treatment effect (*rmstD*(*t* ^*^) = 0). The grey area corresponds to the pointwise 95 % confidence interval. MAC-NPC: Meta-Analysis of Chemotherapy in Nasopharynx Carcinoma; rmstD: difference in restricted mean survival time

The overall proportional hazards assumption was verified at the 5 % significance level (*p* = 0.09) according to the methodology described by Wei et al*.* [6], in which trial-specific *p*-values from Grambsch-Therneau test [36] are pooled. The $rmstD\left({t}^{\ast}\right)$ ranged from 0.17 to 0.23 years at ${t}^{\ast }$= 5 years and from 0.46 to 0.55 years at ${t}^{\ast }$= 10 years across the estimation methods (Table 3). For Pooled Kaplan-Meier and Pooled Exponential using a random effects model, the $rmstD\left({t}^{\ast}\right)$ was not significantly different from 0. As there was high treatment effect heterogeneity in the MAC-NPC, a DerSimonian-Laird random effects model was deemed more appropriate to aggregate the trial-specific ${rmstD}_j\left({t}^{\ast}\right)$. As previously seen in the simulation study, a fixed effect model would underestimate the variance of the overall estimate. Also, similarly to our simulation study with proportional hazards, larger values for $rmstD\left({t}^{\ast}\right)$ and SE ($rmstD\left({t}^{\ast}\right)$) were found at ${t}^{\ast }$= 5 years as compared to at ${t}^{\ast }$= 10 years. Figure 3 displays the forest plot for trial-specific ${rmstD}_j\left({t}^{\ast}\right)$ and overall $rmstD\left({t}^{\ast}\right)$ estimated using Pooled Kaplan-Meier with DerSimonian-Laird random effects at ${t}^{\ast }$= 10 for the MAC-NPC meta-analysis. Figure 4 displays the overall *rmstD*(t^*^) estimated by Pooled Kaplan-Meier with DerSimonian-Laird random effects when varying ${t}^{\ast }$; it shows that the $rmstD\left({t}^{\ast}\right)$ is not significantly different from 0 for ${t}^{\ast }$ϵ [0;10] years. The same graphic for the overall $rmstD\left({t}^{\ast}\right)$ is displayed as Additional file 1: Figure S1.

**Table 3:** Results for comparisons of methods in estimating the difference in restricted mean survival time (*rmstD*) in MAC-NPC and MAC-NPC2 meta-analyses

Meta-analysis model	Methods	MAC-NPC					MAC-NPC2						
		*t* ^*^ = 5 years			*t* ^*^ = 10 years			*t* ^*^ = 5 years			*t* ^*^ = 10 years		
		*rmstD*	SE	*p*-value	*rmstD*	SE	*p*-value	*rmstD*	SE	*p*-value	*rmstD*	SE	*p*-value
	Naïve Kaplan-Meier	0.20	0.08	0.008	0.51	0.19	0.006	0.17	0.04	<0.001	0.54	0.11	<0.001
Random effects	Pooled Kaplan-Meier	0.17	0.11	0.106	0.49	0.28	0.081	0.20	0.05	<0.001	0.59	0.13	<0.001
	Pooled Exponential	0.17	0.09	0.076	0.51	0.29	0.078	0.17	0.03	<0.001	0.55	0.11	<0.001
	Peto-quintile	0.23	0.09	0.007	0.55	0.22	0.011	0.21	0.04	<0.001	0.59	0.12	<0.001
Fixed effect	Pooled Kaplan-Meier	0.20	0.07	0.005	0.52	0.18	0.004	0.18	0.04	<0.001	0.59	0.10	<0.001
	Pooled Exponential	0.18	0.06	0.003	0.55	0.18	0.002	0.17	0.03	<0.001	0.56	0.09	<0.001
	Peto-quintile	0.20	0.07	0.006	0.46	0.16	0.004	0.18	0.04	<0.001	0.53	0.09	<0.001

*MAC-NPC* meta-analysis of chemotherapy in nasopharynx carcinoma, *rmstD* difference in restricted mean survival time, *SE* standard error, *t* ^*^ time horizon

### Update of Meta-Analysis of Chemotherapy in Nasopharynx Carcinoma (MAC-NPC2)

The MAC-NPC2 [35], the update of the MAC-NPC, included new trials as well as updated follow-up for trials included in the MAC-NPC (*N* = 5,028 patients within 23 comparisons). For overall survival, a significant pooled HR of 0.79 (95 % CI: [0.73;0.86], *p* < 0.001) in favor of CT + RT was obtained with a fixed effect model. In the MAC-NPC2, there was less evidence of treatment effect heterogeneity (Q-test: *p* = 0.09; Higgins’ I^2^ = 30 %) than in the MAC-NPC. The pooled HR with a DerSimonian-Laird random effects model [28] was 0.79 (95 % CI: [0.70;0.87], *p* <0.001).

The pooled *p*-value test (*p* = 0.16) suggested that the overall proportional hazards assumption was appropriate. The $rmstD\left({t}^{\ast}\right)$ ranged from 0.17 to 0.21 years at ${t}^{\ast }$= 5 years and from 0.53 to 0.59 years at ${t}^{\ast }$= 10 across the estimation methods (Table 3). The $rmstD\left({t}^{\ast}\right)$ was significantly different from 0 and in favor of the RT + CT arm with all of the methods. As compared to the results in the MAC-NPC, the standard error of the $rmstD\left({t}^{\ast}\right)$ was lower in the MAC-NPC2 with a $rmstD\left({t}^{\ast}\right)$ of similar magnitude for all the methods. This was consistent with the simulation results, as there were more trials and overall more patients included in the MAC-NPC2. The forest plot for the MAC-NPC2 displaying trial-specific ${rmstD}_j\left({t}^{\ast}\right)$ and the overall $rmstD\left({t}^{\ast}\right)$ at ${t}^{\ast }$= 10 years estimated using the Pooled Kaplan-Meier method with DerSimonian-Laird random effects is provided in Additional file 1: Figure S2.

## Discussion

The difference in restricted mean survival time ($rmstD\left({t}^{\ast}\right)$) is an appealing alternative to the hazard ratio (HR) as measure of treatment effect, because it does not require the proportional hazards assumption and is considered to have a more intuitive interpretation [3, 5, 6]. Furthermore, the $rmstD\left({t}^{\ast}\right)$ is directly related to cost-effectiveness analysis as it is the denominator of the incremental cost-effectiveness ratio, so one can use the $rmstD\left({t}^{\ast}\right)$ estimation from a previous publication to perform a cost-effectiveness analysis. We previously showed that in a cost-effectiveness analysis even small variations in the estimate of the $rmstD\left({t}^{\ast}\right)$ from an individual patient data (IPD) meta-analysis can yield significantly different reimbursement conclusions [37]. However, to our knowledge only one evaluation of the methods to estimate the $rmstD\left({t}^{\ast}\right)$ from IPD meta-analysis is available to date [6].

In this study, we compared different methods to estimate the $rmstD\left({t}^{\ast}\right)$ from IPD meta-analysis in different scenarios varying several key meta-analysis parameters. We showed that Pooled Kaplan-Meier was rarely biased. Similarly, Naïve Kaplan-Meier was unbiased in all scenarios, whereas Pooled Exponential showed a bias with non-proportional hazards at ${t}^{\ast }$= 10 years and an even larger bias at t^*^ = 5 years. Peto-quintile underestimated the $rmstD\left({t}^{\ast}\right)$, except with non-proportional hazards at ${t}^{\ast }$= 5 years. In case of treatment effect heterogeneity, the use of a fixed effect model was not appropriate and all methods except Pooled Kaplan-Meier and Pooled Exponential with DerSimonian-Laird random effects underestimated the standard error of the $rmstD\left({t}^{\ast}\right)$. Overall, the Pooled Kaplan-Meier method with DerSimonian-Laird random effects formed the best compromise in terms of bias and variance for estimating the $rmstD\left({t}^{\ast}\right)$ from IPD meta-analysis.

In the IPD meta-analyses studying the effect of chemotherapy (CT) plus radiotherapy (RT) versus RT alone in nasopharynx carcinoma, the $rmstD\left({t}^{\ast },=,10,\kern0.5em ,\mathrm{years}\right)$ estimated using the Pooled Kaplan-Meier method with DerSimonian-Laird random effects was 0.49 years (95 % CI: [−0.06;1.03], *p* = 0.08) in the MAC-NPC [34] and 0.59 years (95 % CI: [0.34;0.84], *p* < 0.0001) in its updated version MAC-NPC2 [35]. In other words, the addition of CT to RT extended the 10-year mean survival time by 7.1 months (95 % CI 4.1;10.1) in MAC-NPC2. We believe the clinical interpretation with the $rmstD\left({t}^{\ast}\right)$ is more intuitive than the one derived from the pooled hazard ratio with DerSimonian-Laird random effects of 0.79 (95 % CI 0.70−0.87) in MAC-NPC2.

The $rmstD\left({t}^{\ast}\right)$ is an absolute outcome measure which depends both on the baseline hazard and on the relative treatment effect. Consequently, the heterogeneity test when pooling the ${rmstD}_j\left({t}^{\ast}\right)$ reflects both baseline hazard and relative treatment effect heterogeneities. Deeks already showed that in 551 systematic reviews with binary outcomes the heterogeneity was higher for an absolute outcome than for a relative outcome [38]. In our IPD meta-analyses in nasopharynx carcinoma, the heterogeneity was slightly higher when pooling the ${rmstD}_j\left({t}^{\ast}\right)$ with Pooled Kaplan-Meier than when pooling the hazard ratios: for the MAC-NPC there was a small increase in the heterogeneity with Cochran Q test *p*-value = 0.03, I^2^ = 50 % for HR as compared to *p* = 0.02, I^2^ = 54 % for $rmstD\left({t}^{\ast },=,10,\kern0.5em ,\mathrm{years}\right)$ (Fig. 3). For the MAC-NPC2, this increase was more pronounced with *p* = 0.09, I^2^ = 30 % for HR versus *p* = 0.01, I^2^ = 45 % for $rmstD\left({t}^{\ast },=,10,\kern0.5em ,\mathrm{years}\right)$ (Additional file 1: Figure S1). Wei and colleagues showed a similar trend in their second example (*p* = 0.47, I^2^ = 0 % for HR and *p* = 0.20, I^2^ = 24 % for $rmstD\left({t}^{\ast },=,5,\kern0.5em ,\mathrm{years}\right)$) [6].

In our simulation study, we have induced between-trial heterogeneity for the baseline hazard and for the treatment effect using two random effects. As a matter of fact, both random effects can be tested, by testing Var(*A*_*j*_) = *σ*^*2*^ = 0 and Var(*B*_*j*_) = *τ*^*2*^ = 0. Testing for the presence of treatment effect heterogeneity (*τ*^*2*^ = 0) corresponds to the Cochran Q-test which we have used in the MAC-NPC applications [34, 35]. Commenges and Andersen [39] and Biard and colleagues [40] proposed respectively the use of score tests or permutation tests for testing the baseline heterogeneity (*σ*^*2*^ = 0) in proportional hazard models. Rondeau et al. [19] tested both the baseline hazard (*σ*^*2*^ = 0) and the treatment effect heterogeneity between trials (*τ*^*2*^ = 0) using a mixture of *χ*^2^ distributions in one-stage Cox models.

Recent techniques like the one proposed by Guyot et al*.* [41] allow one to reconstruct IPD based on published Kaplan-Meier curves, which could be useful to recalculate the $rmstD\left({t}^{\ast}\right)$ even for aggregate data. However, we suggest that clinical publications for single (multicenter) clinical trial or IPD meta-analysis should report the $rmstD\left({t}^{\ast}\right)$ at different time horizons ${t}^{\ast }$ of clinical interest in addition to the hazard ratio. This way the $rmstD\left({t}^{\ast}\right)$ would be available for future economic evaluations. This is of particular relevance as a previous study stated that the survival outcome in a cost-effectiveness analysis based on a clinical trial or a meta-analysis should be estimated with the same statistical model used for efficacy [42].

Among the two-stage methods studied by Wei et al*.*, the non-parametric pseudo-values method was disregarded, as Wei et al. showed that it led to similar results as the non-parametric Pooled Kaplan-Meier method [6]. Also, among parametric models we chose the exponential model instead of the Royston and Parmar flexible parametric model for ease of computation. In addition, we chose to study other non-parametric methods from the medical literature that have been actually applied in practice. Parametric methods developed for network meta-analysis were not included [43, 44]. Furthermore, methods using the percentile ratio [45, 46] were beyond the scope of this study, which focused on the $rmstD\left({t}^{\ast}\right)$. In addition, in this simulation study, we only considered balanced trials and we did not vary the administrative censoring rate.

The $rmstD\left({t}^{\ast}\right)$ is inherently dependent on the choice of ${t}^{\ast }$. Also, we showed that its standard error gets larger as ${t}^{\ast }$increases (Fig. 1). Karrison recommended to choose a maximum time horizon ${t}^{\ast }$ such that SE(*S*(${t}^{\ast }$)) is less than a chosen ceiling value [29, 47]. In the particular case of an IPD meta-analysis, trials can have different lengths of follow-up, and there is thus a compromise to achieve between small values of ${t}^{\ast }$ that censor a lot of data with a high loss of information, and high values of ${t}^{\ast }$ that need a massive use of extrapolation. Wei and colleagues stated that the choice of ${t}^{\ast }$ should also be of clinical interest, and they suggested plotting the $rmstD\left({t}^{\ast}\right)$ against ${t}^{\ast }$ to see how the treatment effect varies over time. In MAC-NPC for instance such a plot shows that the $rmstD\left({t}^{\ast}\right)$ was not significantly different from 0 with ${t}^{\ast }$ϵ [0;10] years based on pointwise confidence intervals (Fig. 4). In two recent papers, Tian et al. [48] and Zhao et al. [4] have proposed a simultaneous confidence interval of the $rmstD\left({t}^{\ast}\right)$ in the context of one randomized controlled trial. However, an extension to the context of IPD meta-analyses or multicenter clinical trials has not yet been proposed and may be the subject of further research.

Depending on the choice of the time horizon ${t}^{\ast }$, some trials included in the IPD meta-analysis may have a follow-up not long enough to reach ${t}^{\ast }$. In our study, for such trials, we used the extrapolation method proposed by Brown et al*.* [32] until ${t}^{\ast }$ for the Naïve Kaplan-Meier and the Pooled Kaplan-Meier methods. Lamb and colleagues [10] have shown that this extrapolation method is less biased than the mean survival time restricted at the last observed event time. For lifetime extrapolation, which can be needed in cost-effectiveness analysis, one can estimate the difference in mean survival time using the Pooled Kaplan-Meier with a DerSimonian-Laird random effects model. In each trial, the difference in mean survival time would be estimated using Kaplan-Meier curves with extrapolated parametric tails [9, 10]. Similarly, for the two other non-parametric methods Naïve Kaplan-Meier and Peto-quintile, one can extrapolate the survival curves beyond the last observed failure time by using an extrapolated parametric tail.

## Conclusions

The difference in restricted mean survival time ($rmstD\left({t}^{\ast}\right)$) is an appealing alternative to the hazard ratio to measure the treatment effect in a meta-analysis of time-to-event outcomes, as it is free of the proportional hazards assumption and its interpretation is more intuitive. We compared methods to estimate the $rmstD\left({t}^{\ast}\right)$ from an individual patient data meta-analysis. In our simulation study, in which a large panel of meta-analysis parameters was varied, the two-stage Pooled Kaplan-Meier method with DerSimonian-Laird random effects formed the best compromise in terms of bias and variance. Thus, Pooled Kaplan-Meier with DerSimonian-Laird random effects should be the preferred method to estimate the difference in restricted mean survival time from an individual patient data meta-analysis or from a multicenter clinical trial.

### Availability of data and materials

Data were used with permission obtained from the MAC-NPC Collaborative Group investigators, who agreed to share their data with us by signing an amendment to the original protocol. The French data protection authority (CNIL - Commission Nationale de l’Informatique et des Libertés) does not allow us to make these data publicly available.

## Additional file

Additional file 1: Supplementary statistical details, **Tables S1-S7** and **Figures S1-S2**. (DOCX 339 kb)

## Abbreviations

ASE: average standard error
CI: confidence interval
CT: chemotherapy
ESE: empirical standard error
HR: hazard ratio
IPD: individual patient data
MAC-NPC: Meta-Analysis of Chemotherapy in Nasopharynx Carcinoma
RMST: restricted mean survival time
rmstD: difference in restricted mean survival time RT radiotherapy
RT: radiotherapy

## References

[1] Irwin JO (1949). The standard error of an estimate of expectation of life, with special reference to the expectation of tumour less life in experiments with mice. J Hygiene.

[2] Andersen PK, Hansen MG, Klein JP (2004). Regression analysis of restricted mean survival time based on pseudo-observations. Lifetime Data Anal.

[3] Royston P, Parmar MK (2013). Restricted mean survival time: an alternative to the hazard ratio for the design and analysis of randomized trials with a time-to-event outcome. BMC Med Res Methodol.

[4] Zhao L, Claggett B, Tian L, Uno H, Pfeffer MA, Solomon SD (2015). On the restricted mean survival time curve in survival analysis. Biometrics.

[5] Royston P, Parmar MK (2011). The use of restricted mean survival time to estimate the treatment effect in randomized clinical trials when the proportional hazards assumption is in doubt. Stat Med.

[6] Wei Y, Royston P, Tierney JF, Parmar MK (2015). Meta-analysis of time-to-event outcomes from randomized trials using restricted mean survival time: application to individual participant data. Stat Med.

[7] Jackson CH, Sharples LD, Thompson SG (2010). Survival models in health economic evaluations: balancing fit and parsimony to improve prediction. Int J Biostat.

[8] Latimer N (2013). Survival analysis for economic evaluations alongside clinical trials--extrapolation with patient-level data: inconsistencies, limitations, and a practical guide. Med Decis Making.

[9] Gong Q, Fang L (2012). Asymptotic properties of mean survival estimate based on the Kaplan-Meier curve with an extrapolated tail. Pharm Stat.

[10] Lamb KE, Williamson EJ, Coory M, Carlin JB (2015). Bias and precision of measures of survival gain from right-censored data. Pharm Stat.

[11] Altman DG, Deeks JJ (2002). Meta-analysis, Simpson’s paradox, and the number needed to treat. BMC Med Res Methodol.

[12] Cates CJ (2002). Simpson’s paradox and calculation of number needed to treat from meta-analysis. BMC Med Res Methodol.

[13] Rücker G, Schumacher M (2008). Simpson’s paradox visualized: the example of the rosiglitazone meta-analysis. BMC Med Res Methodol.

[14] Glidden DV, Vittinghoff E (2004). Modelling clustered survival data from multicentre clinical trials. Stat Med.

[15] Legrand C, Ducrocq V, Janssen P, Sylvester R, Duchateau L (2005). A Bayesian approach to jointly estimate centre and treatment by centre heterogeneity in a proportional hazards model. Stat Med.

[16] Munda M, Legrand C (2014). Adjusting for centre heterogeneity in multicentre clinical trials with a time-to-event outcome. Pharm Stat.

[17] Michiels S, Baujat B, Mahé C, Sargent DJ, Pignon JP (2005). Random effects survival models gave a better understanding of heterogeneity in individual patient data meta-analyses. J Clin Epidemiol.

[18] Smith CT, Williamson PR, Marson AG (2005). Investigating heterogeneity in an individual patient data meta-analysis of time to event outcomes. Stat Med.

[19] Rondeau V, Michiels S, Liquet B, Pignon JP (2008). Investigating trial and treatment heterogeneity in an individual patient data meta-analysis of survival data by means of the penalized maximum likelihood approach. Stat Med.

[20] Stewart GB, Altman DG, Askie LM, Duley L, Simmonds MC, Stewart L (2012). Statistical analysis of individual participant data meta-analyses: a comparison of methods and recommendations for practice. PLoS One.

[21] Bowden J, Tierney JF, Simmonds M, Copas AJ, Higgins JP (2011). Individual patient data meta-analysis of time-to-event outcomes: one-stage versus two-stage approaches for estimating the hazard ratio under a random effects model. Res Synth Methods.

[22] Smith CT, Williamson PR (2007). A comparison of methods for fixed effects meta-analysis of individual patient data with time to event outcomes. Clin Trials.

[23] Andersen PK, Perme MP (2010). Pseudo-observations in survival analysis. Stat Methods Med Res.

[24] Royston P, Parmar MK (2002). Flexible parametric proportional-hazards and proportional-odds models for censored survival data, with application to prognostic modelling and estimation of treatment effects. Stat Med.

[25] Early Breast Cancer Trialists’ Collaborative Group (1990). Treatment of early breast cancer vol.1: worldwide evidence 1985–1990.

[26] Pignon JP, Le Maître A, Maillard E, Bourhis J, on behalf of the MACH-NC Collaborative Group (2009). Meta-analysis of chemotherapy in head & neck cancer (MACH-NC): an update on 93 randomized trials and 17 346 patients. Radiother Oncol.

[27] Peto R, Davies C, Early Breast Cancer Trialists’ Collaborative Group (2012). Comparisons between different polychemotherapy regimens for early breast cancer: meta-analyses of long-term outcome among 100,000 women in 123 randomised trials. Lancet.

[28] DerSimonian R, Laird N (1986). Meta-analysis in clinical trials. Control Clin Trials.

[29] Karrison T (1997). Use of Irwin’s restricted mean as an index for comparing survival in different treatment groups—interpretation and power considerations. Control Clin Trials.

[30] Durand-Zaleski I, Roche B, Buyse M, Carlson R, O’Connell MJ, Rougier P (1997). Economic implications of hepatic arterial infusion chemotherapy in treatment of nonresectable colorectal liver metastases. J Natl Cancer Inst.

[31] Rotolo F, Michiels S (2014). Testing the treatment effect on competing causes of death in oncology clinical trials. BMC Med Res Methodol..

[32] Brown J, Hollander M, Korwar R. Nonparametric tests of independence for censored data with application to heart transplant studies. *Reliability and Biometry, Statistical Analysis of Lifelength* 1974;327–54

[33] Burton A, Altman DG, Royston P, Holderal RL (2006). The design of simulation studies in medical statistics. Stat Med.

[34] Baujat B, Audry H, Bourhis J, Chan A, Onat H, Chua D (2006). Chemotherapy in locally advanced nasopharyngeal carcinoma: an individual patient data meta-analysis of eight randomized trials and 1753 patients. Int J Radiat Oncol Biol Phys.

[35] Blanchard P, Lee A, Marguet S, Leclercq J, Ng WT, Ma J (2015). Chemotherapy and radiotherapy in nasopharyngeal carcinoma: an update of the MAC-NPC meta-analysis. Lancet Oncol.

[36] Grambsch P, Therneau TM (1994). Proportional hazards tests and diagnostics based on weighted residuals. Biometrika.

[37] Lueza B, Mauguen A, Pignon J-P, Rivero-Arias O, Bonastre J (2016). Difference in restricted mean survival time for cost-effectiveness analysis using individual patient data meta-analysis: evidence from a case study. PLoS One.

[38] Deeks JJ (2002). Issues in the selection of a summary statistic for meta-analysis of clinical trials with binary outcomes. Stat Med.

[39] Commenges D, Andersen PK (1995). Score test of homogeneity for survival data. Lifetime Data Anal.

[40] Biard L, Porcher R, Resche-Rigon M (2014). Permutation tests for centre effect on survival endpoints with application in an acute myeloid leukaemia multicentre study. Stat Med.

[41] Guyot P, Ades A, Ouwens MJ, Welton NJ (2012). Enhanced secondary analysis of survival data: reconstructing the data from published Kaplan-Meier survival curves. BMC Med Res Methodol.

[42] Guyot P, Welton NJ, Ouwens MJNM, Adesa E (2011). Survival time outcomes in randomized, controlled trials and meta-analyses: the parallel universes of efficacy and cost-effectiveness. Value Health.

[43] Jansen JP (2011). Network meta-analysis of survival data with fractional polynomials. BMC Med Res Methodol.

[44] Ouwens MJNM, Philips Z, Jansen JP (2010). Network meta-analysis of parametric survival curves. Res Synth Methods.

[45] Siannis F, Barrett JK, Farewell VT, Tierney JF (2010). One-stage parametric meta-analysis of time-to-event outcomes. Stat Med.

[46] Barrett JK, Farewell VT, Siannis F, Tierney J, Higgins JPT (2012). Two-stage meta-analysis of survival data from individual participants using percentile ratios. Stat Med.

[47] Karrison T (1987). Restricted mean life with adjustment for covariates. J Am Stat Assoc.

[48] Tian L, Zhao L, Wei LJ (2014). Predicting the restricted mean event time with the subject’s baseline covariates in survival analysis. Biostatistics.

