# Erratum to: Bias and precision of methods for estimating the difference in restricted mean survival time from an individual patient data meta-analysis

**DOI:** 10.1186/s12874-016-0154-y

**Published:** 2016-06-15

**Authors:** Béranger Lueza, Federico Rotolo, Julia Bonastre, Jean-Pierre Pignon, Stefan Michiels

**Affiliations:** Gustave Roussy, Université Paris-Saclay, Service de biostatistique et d’épidémiologie, Villejuif, F-94805 France; Université Paris-Saclay, Univ. Paris-Sud, UVSQ, CESP, INSERM, Villejuif, F-94085 France; Ligue Nationale Contre le Cancer meta-analysis platform, Gustave Roussy, Villejuif, F-94085 France

## Erratum

After publication of the original article [1], it came to the authors’ attention that there were errors affecting the equations and Table 3. These corrections were mentioned by the authors during the proofing process, but unfortunately not implemented before final publication.

**Table 3:** Results for comparisons of methods in estimating the difference in restricted mean survival time (*rmstD*) in MAC-NPC and MAC-NPC2 meta-analyses

Meta-analysis model	Methods	MAC-NPC						MAC-NPC2					
		*t* ^*^ = 5 years			*t* ^*^ = 10 years			*t* ^*^ = 5 years			*t* ^*^ = 10 years		
		*rmstD*	SE	*p*-value	*rmstD*	SE	*p*-value	*rmstD*	SE	*p*-value	*rmstD*	SE	*p*-value
	Naïve Kaplan-Meier	0.20	0.08	0.008	0.51	0.19	0.006	0.17	0.04	<0.001	0.54	0.11	<0.001
Random effects	Pooled Kaplan-Meier	0.17	0.11	0.106	0.49	0.28	0.081	0.20	0.05	<0.001	0.59	0.13	<0.001
	Pooled Exponential	0.17	0.09	0.076	0.51	0.29	0.078	0.17	0.03	<0.001	0.55	0.11	<0.001
	Peto-quintile	0.23	0.09	0.007	0.55	0.22	0.011	0.21	0.04	<0.001	0.59	0.12	<0.001
Fixed effect	Pooled Kaplan-Meier	0.20	0.07	0.005	0.52	0.18	0.004	0.18	0.04	<0.001	0.59	0.10	<0.001
	Pooled Exponential	0.18	0.06	0.003	0.55	0.18	0.002	0.17	0.03	<0.001	0.56	0.09	<0.001
	Peto-quintile	0.20	0.07	0.006	0.46	0.16	0.004	0.18	0.04	<0.001	0.53	0.09	<0.001

*MAC-NPC* Meta-Analysis of Chemotherapy in Nasopharynx Carcinoma, *rmstD* difference in restricted mean survival time, *SE* standard error, *t* ^*^ time horizon

Equations 9–11, 14, 18 and 20 were published containing minor typographical errors, affecting subscript characters, special characters and line breaks. These equations have been updated in the original article and, accordingly, references to these equations within the main text have been corrected also.

In Table 3, the distribution of the columns was incorrect. The ‘MAC-NPC’ and ‘MAC-NPC2’ column headings should each have been over two sets of “t^*^ = 5 years” and “t^*^ = 10 years” sub-headings. In turn, these sub-headings should have contained 6 columns; the ‘MAC-NPC2’ heading contained 7, an extra ‘*rmstD*’ column. This has been corrected in the original article.

In addition, the authors noticed an error in Figure 3 which was not submitted at proofing. The vertical dashed line should have crossed the black diamond, representing the overall rmstD, through the center. A correct version of Fig. 3 is now present in the original manuscript and is published in this erratum.

**Fig. 3:**
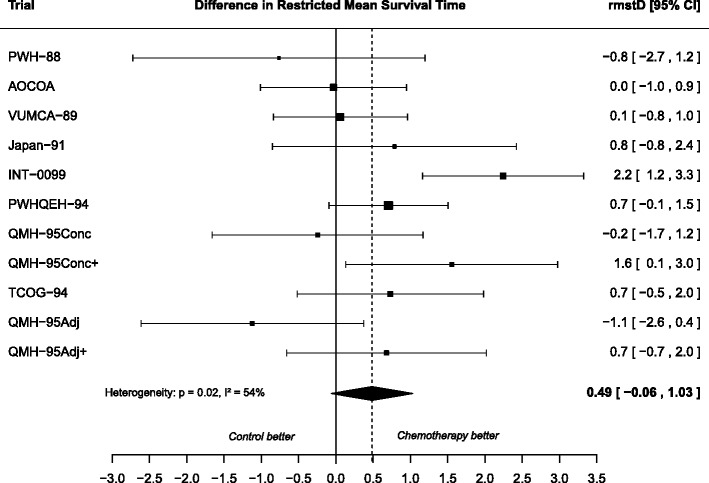
Forest plot for differences in restricted mean survival time estimated at 10 years using the Pooled Kaplan-Meier method with random effects applied to the MAC-NPC meta-analysis. Each trial is represented by a square, the center of which denotes the difference in restricted mean survival time (rmstD) for that trial comparison, with the horizontal lines showing the 95 % confidence intervals (CI). The size of the square is directly proportional to the amount of information contributed by the trial. The diamond represents the overall rmstD, with the center denoting the rmstD and the extremities the 95 % CI. The rmstDs are expressed in year
