# Supplementary material for: Bias and precision of methods for estimating the difference in restricted mean survival time from an individual patient data meta-analysis
Source: BMC Med Res Methodol. 2016 Mar 29;16:37. doi: 10.1186/s12874-016-0137-z (PMC4812643; doi:10.1186/s12874-016-0137-z)
Supplement: Additional file 1: — Supplementary statistical details, Tables S1-S7 and Figures S1-S2. (DOCX 339 kb) [file 12874_2016_137_MOESM1_ESM.docx]

**Supplementary statistical details**

**Fixed effect and random effects meta-analysis models**

A pooled estimate of the treatment effect $\hat{\theta}_{pooled}$ is obtained by aggregating the estimated treatment effects across *J* trials using either fixed effect or random effects weights [1]:

$\hat{\theta}_{pooled}=\frac{\sum_{j=1}^{J} \hat{\theta}_{j}w_{j}}{\sum_{j=1}^{J} w_{j}}$ and $\hat{Var}[\theta_{pooled}]=\frac{1}{\sum_{j=1}^{J} w_{j}}$ (A1)

where weights are estimated as $w_{j}=\frac{1}{\hat{Var}(\hat{\theta}_{j})}$ for fixed effect, and as $w_{j}=\frac{1}{\hat{Var}\left( \hat{\theta}_{j} \right)+\tau^{2}}$ for random effects. The parameter $\tau^{2}$ accounts for between-trial treatment effect heterogeneity of $\hat{\theta}_{j}$ and is estimated using the DerSimonian-Laird method of moments [2].

In the investigated methods, $\hat{\theta}_{j}=\log(\hat{HR_{j}})$ for the Peto-quintile method, and $\hat{\theta}_{j}={rmstD}_{j}(t^{*})$ for the Pooled Kaplan-Meier and the Pooled Exponential methods.

**Estimation of the variance of the rmstD(t^*^)**

The variance of the difference in restricted mean survival time (*rmstD*(*t^*^*)) can be estimated as [3]

$\hat{Var}(rmstD\left( t^{*} \right))=\hat{Var}\left( RMST_{1}\left( t^{*} \right) \right)+\hat{Var}({RMST}_{0}\left( t^{*} \right))$ (A2)

We used the delta method, based on the Taylor series approximation (Appendix 1 from [4]), to derive a variance estimator for the *RMST*(*t^*^*) for all the methods but Peto-quintile:

$Var\left[ f\left( X \right) \right]=Var\left[ X \right]\left[ \frac{\partial f(X)}{\partial X} \right]^{2}$ (A3)

Further details are given below for the Naïve Kaplan-Meier and Pooled Kaplan-Meier methods, and for the Pooled Exponential method.

1. Naïve Kaplan-Meier and Pooled Kaplan-Meier

Based on the delta method (A3), the Greenwood’s formula for the variance of the Kaplan-Meier survival function is [4]

$\hat{Var}\left( \hat{S}\left( t \right) \right)=\left[ \hat{S}\left( t \right) \right]^{2}\sum_{t_{i}\leq t_{D}} \frac{d_{i}}{Y_{i}(Y_{i}-d_{i})}$ (A4)

with *D* the number of distinct event times (*t_1_*< *t_2_* < …< *t_D_*), *Y_i_* the number of individuals who are at risk at time *t_i_* and *d_i_* the number of events at time *t_i_*.

To estimate the variance of the restricted mean survival time in each arm, we used the following formula which derives from Greenwood’s formula (A4) [4,5]:

$\hat{Var}\left( RMST\left( t^{*} \right) \right)=\sum_{i=1}^{D} \left[ \int_{t_{i}}^{t^{*}} \hat{S}\left( t \right)dt \right]^{2}\frac{d_{i}}{Y_{i}(Y_{i}-d_{i})}$ (A5)

Replacing $\hat{S}\left( t \right)$ by the Kaplan-Meier estimator in (A5), it led to (page 55 in [4]):

$\hat{Var}\left( RMST_{arm}\left( t^{*} \right) \right)=\frac{m}{m-1}\sum_{i=1}^{D-1} \frac{a_{i}^{2}}{Y_{i}(Y_{i}-d_{i})}$ (A6)

where $a_{i}=\sum_{l=i}^{D-1} \hat{S}(t_{l})(t_{l+1}-t_{l})$ and $m=\sum_{l=1}^{D} d_{l}$

1. Pooled Exponential

For the Pooled Exponential method, based on the equation (7) in the manuscript, the variance of the *RMST*(*t^*^*) in each trial *j* and in each arm is

$\hat{Var}\left[ RMST_{j,arm}\left( t^{*} \right) \right]=Var\left[ \frac{1-e^{-\hat{\lambda}_{j,arm}t^{*}}}{\hat{\lambda}_{j,arm}} \right]$ (A7)

where $\hat{\lambda}_{j,arm}$ is the maximum likelihood estimate of the scale parameter for the exponential distribution for the experimental arm and the control arm (noted 1 and 0)

By direct application of the delta method in (A7), the variance of *RMST_j,arm_*(*t^*^*) is

$\hat{Var}\left[ RMST_{j,arm}\left( t^{*} \right) \right]=\hat{Var}\left[ \hat{\lambda}_{j,arm} \right]\left[ \frac{\partial f\left( \hat{\lambda}_{j,arm} \right)}{\partial\hat{\lambda}_{j,arm}} \right]^{2}$ (A8)

From this we can obtain an easily computable estimator of the variance:

$\hat{Var}\left[ RMST_{j,arm}\left( t^{*} \right) \right]=\hat{Var}\left[ \hat{\lambda}_{j,arm} \right]\left[ \frac{{\hat{\lambda}_{j,arm}\times t^{*}\times e}^{-\hat{\lambda}_{j,arm}t^{*}}-\left( 1-e^{-\hat{\lambda}_{j,arm}t^{*}} \right)}{{\hat{\lambda}_{j,arm}}^{2}} \right]^{2}$ (A9)

References:

1. Whitehead A. Meta-analysis of Controlled Clinical Trials. Wiley: Chichester, 2002.

2. DerSimonian R, Laird N. Meta-analysis in clinical trials. *Control Clin Trials* 1986; 7: 177–88.

3. Karrison T. Restricted mean life with adjustment for covariates. J Am Stat Assoc 1987; 82: 1169–76.

4. Hosmer DW, Lemeshow S, May S. Applied Survival Analysis: regression modeling of time-to-event data, 2nd ed. New York: John Wiley & Sons, 2008.

5. Klein JP, Moeschberger ML. Survival Analysis: techniques for censored and truncated data, 1st ed. New York: Springer-Verlag, 1997.

**Table S1. Simulation results for comparisons of methods in estimating the difference in restricted mean survival time (rmstD). Scenario with 5 trials and 200 patients per trial and with proportional hazards.**

| **Heterogeneity**  **scenario** | **Methods** | $\boldsymbol{\beta}$ **= 0** | | | | | | | | $\boldsymbol{\beta}$ **= -0.2** | | | | | | | | $\boldsymbol{\beta}$ **= -0.7** | | | | | | |
| --- | --- | --- | --- | --- | --- | --- | --- | --- | --- | --- | --- | --- | --- | --- | --- | --- | --- | --- | --- | --- | --- | --- | --- | --- |
|  |  | **t* = 5**  **True *rmstD* = 0** | | | | **t* = 10**  **True *rmstD* = 0** | | | | **t* = 5**  **True *rmstD* = 0.2** | | | | **t* = 10**  **True *rmstD* = 0.6** | | | | **t* = 5**  **True *rmstD* = 0.8** | | | | **t* = 10**  **True *rmstD* = 2.0** | | |
|  |  | Bias | ESE | ASE |  | Bias | ESE | ASE |  | Bias | ESE | ASE |  | Bias | ESE | ASE |  | Bias | ESE | ASE |  | Bias | ESE | ASE |
| **(σ²,τ²) = (0.01;0.01)** | **Naïve Kaplan-Meier** | 0.00 | 0.12 | 0.11 |  | 0.00 | 0.28 | 0.24 |  | 0.00 | 0.12 | 0.11 |  | 0.00 | 0.29 | 0.24 |  | 0.01 | 0.12 | 0.11 |  | 0.01 | 0.28 | 0.23 |
|  | **Pooled Kaplan-Meier** | 0.00 | 0.12 | 0.13 |  | 0.00 | 0.29 | 0.30 |  | 0.00 | 0.12 | 0.13 |  | 0.01 | 0.31 | 0.30 |  | 0.01 | 0.12 | 0.13 |  | 0.02 | 0.30 | 0.29 |
|  | **Pooled Exponential** | 0.00 | 0.10 | 0.11 |  | 0.00 | 0.27 | 0.28 |  | 0.00 | 0.11 | 0.11 |  | 0.00 | 0.28 | 0.28 |  | 0.01 | 0.11 | 0.11 |  | 0.02 | 0.27 | 0.27 |
|  | **Peto-quintile** | 0.00 | 0.11 | 0.10 |  | 0.00 | 0.24 | 0.21 |  | -0.01 | 0.12 | 0.10 |  | -0.07 | 0.25 | 0.21 |  | -0.04 | 0.12 | 0.10 |  | -0.21 | 0.26 | 0.23 |
| **(σ²,τ²) = (0.01;0.10)** | **Naïve Kaplan-Meier** | 0.00 | 0.19 | 0.11 |  | 0.01 | 0.49 | 0.24 |  | 0.00 | 0.19 | 0.11 |  | 0.01 | 0.48 | 0.24 |  | 0.00 | 0.19 | 0.11 |  | 0.00 | 0.48 | 0.23 |
|  | **Pooled Kaplan-Meier** | 0.00 | 0.20 | 0.18 |  | 0.01 | 0.50 | 0.47 |  | 0.00 | 0.19 | 0.19 |  | 0.00 | 0.49 | 0.47 |  | 0.00 | 0.20 | 0.18 |  | 0.01 | 0.48 | 0.44 |
|  | **Pooled Exponential** | 0.00 | 0.19 | 0.17 |  | 0.01 | 0.48 | 0.45 |  | 0.00 | 0.18 | 0.17 |  | 0.01 | 0.47 | 0.46 |  | 0.00 | 0.19 | 0.17 |  | 0.01 | 0.47 | 0.44 |
|  | **Peto-quintile** | 0.00 | 0.18 | 0.10 |  | 0.00 | 0.43 | 0.22 |  | -0.01 | 0.18 | 0.10 |  | -0.07 | 0.42 | 0.22 |  | -0.04 | 0.19 | 0.10 |  | -0.20 | 0.46 | 0.23 |
| **(σ²,τ²) = (0.10;0.01)** | **Naïve Kaplan-Meier** | -0.01 | 0.12 | 0.11 |  | -0.01 | 0.28 | 0.24 |  | 0.00 | 0.12 | 0.11 |  | 0.01 | 0.27 | 0.24 |  | 0.00 | 0.14 | 0.11 |  | 0.01 | 0.28 | 0.24 |
|  | **Pooled Kaplan-Meier** | -0.01 | 0.12 | 0.13 |  | 0.00 | 0.28 | 0.29 |  | 0.00 | 0.12 | 0.13 |  | 0.00 | 0.29 | 0.29 |  | 0.00 | 0.14 | 0.13 |  | 0.01 | 0.28 | 0.29 |
|  | **Pooled Exponential** | 0.00 | 0.10 | 0.11 |  | -0.01 | 0.26 | 0.28 |  | 0.00 | 0.10 | 0.11 |  | 0.01 | 0.26 | 0.28 |  | 0.00 | 0.12 | 0.12 |  | 0.02 | 0.27 | 0.27 |
|  | **Peto-quintile** | -0.01 | 0.11 | 0.10 |  | -0.01 | 0.23 | 0.22 |  | -0.01 | 0.12 | 0.10 |  | -0.06 | 0.24 | 0.21 |  | -0.03 | 0.13 | 0.11 |  | -0.15 | 0.26 | 0.23 |
| **(σ²,τ²) = (0.10;0.10)** | **Naïve Kaplan-Meier** | 0.00 | 0.18 | 0.11 |  | 0.00 | 0.45 | 0.24 |  | -0.01 | 0.19 | 0.11 |  | 0.00 | 0.48 | 0.24 |  | 0.01 | 0.19 | 0.11 |  | 0.01 | 0.44 | 0.24 |
|  | **Pooled Kaplan-Meier** | 0.00 | 0.18 | 0.18 |  | 0.00 | 0.45 | 0.45 |  | -0.01 | 0.19 | 0.18 |  | 0.00 | 0.47 | 0.45 |  | 0.01 | 0.19 | 0.18 |  | 0.02 | 0.44 | 0.43 |
|  | **Pooled Exponential** | 0.00 | 0.17 | 0.17 |  | 0.00 | 0.44 | 0.44 |  | 0.00 | 0.18 | 0.17 |  | 0.00 | 0.47 | 0.44 |  | 0.01 | 0.19 | 0.18 |  | 0.02 | 0.44 | 0.42 |
|  | **Peto-quintile** | 0.00 | 0.17 | 0.10 |  | 0.00 | 0.40 | 0.22 |  | -0.01 | 0.18 | 0.10 |  | -0.06 | 0.42 | 0.22 |  | -0.02 | 0.19 | 0.11 |  | -0.13 | 0.43 | 0.24 |

A random effects meta-analysis model was used for Pooled Kaplan-Meier, Pooled Exponential and Peto-quintile. Columns in grey (*β* = 0 and *β* = -0.7) are also shown in Table 1 in the main manuscript.

*β*: Size of treatment effect (=log(HR)); *σ²*: baseline hazard heterogeneity; *τ²*: treatment effect heterogeneity; ASE: average standard error; CI: Confidence interval; ESE: empirical standard error; rmstD: difference in restricted mean survival time; *t^*^*: time horizon

| **Heterogeneity**  **scenario** | **Methods** | $\boldsymbol{\beta}$ **= 0** | | | | | | | | $\boldsymbol{\beta}$ **= -0.2** | | | | | | | | $\boldsymbol{\beta}$ **= -0.7** | | | | | | |
| --- | --- | --- | --- | --- | --- | --- | --- | --- | --- | --- | --- | --- | --- | --- | --- | --- | --- | --- | --- | --- | --- | --- | --- | --- |
|  |  | **t* = 5**  **True *rmstD* = 0** | | | | **t* = 10**  **True *rmstD* = 0** | | | | **t* = 5**  **True *rmstD* = 0.2** | | | | **t* = 10**  **True *rmstD* = 0.6** | | | | **t* = 5**  **True *rmstD* = 0.8** | | | | **t* = 10**  **True *rmstD* = 2.0** | | |
|  |  | Bias | ESE | ASE |  | Bias | ESE | ASE |  | Bias | ESE | ASE |  | Bias | ESE | ASE |  | Bias | ESE | ASE |  | Bias | ESE | ASE |
| **(σ²,τ²) = (0.01;0.01)** | **Naïve Kaplan-Meier** | 0.00 | 0.08 | 0.08 |  | 0.00 | 0.18 | 0.17 |  | 0.00 | 0.08 | 0.08 |  | 0.01 | 0.19 | 0.17 |  | 0.00 | 0.08 | 0.08 |  | 0.00 | 0.17 | 0.16 |
|  | **Pooled Kaplan-Meier** | 0.00 | 0.08 | 0.08 |  | 0.00 | 0.20 | 0.20 |  | 0.00 | 0.08 | 0.08 |  | 0.02 | 0.21 | 0.20 |  | 0.01 | 0.08 | 0.08 |  | 0.02 | 0.19 | 0.19 |
|  | **Pooled Exponential** | 0.00 | 0.07 | 0.08 |  | 0.00 | 0.19 | 0.19 |  | 0.01 | 0.07 | 0.08 |  | 0.01 | 0.19 | 0.19 |  | 0.01 | 0.07 | 0.08 |  | 0.03 | 0.17 | 0.18 |
|  | **Peto-quintile** | 0.00 | 0.08 | 0.07 |  | 0.00 | 0.16 | 0.15 |  | -0.01 | 0.08 | 0.07 |  | -0.07 | 0.16 | 0.15 |  | -0.05 | 0.07 | 0.07 |  | -0.22 | 0.16 | 0.16 |
| **(σ²,τ²) = (0.01;0.10)** | **Naïve Kaplan-Meier** | 0.00 | 0.11 | 0.08 |  | 0.01 | 0.27 | 0.17 |  | 0.00 | 0.11 | 0.08 |  | 0.01 | 0.27 | 0.17 |  | 0.00 | 0.11 | 0.08 |  | 0.00 | 0.26 | 0.16 |
|  | **Pooled Kaplan-Meier** | 0.00 | 0.11 | 0.11 |  | 0.01 | 0.28 | 0.28 |  | 0.01 | 0.12 | 0.11 |  | 0.01 | 0.28 | 0.27 |  | 0.01 | 0.11 | 0.11 |  | 0.03 | 0.26 | 0.26 |
|  | **Pooled Exponential** | 0.00 | 0.11 | 0.10 |  | 0.01 | 0.27 | 0.27 |  | 0.01 | 0.11 | 0.10 |  | 0.01 | 0.28 | 0.27 |  | 0.01 | 0.10 | 0.10 |  | 0.03 | 0.26 | 0.26 |
|  | **Peto-quintile** | 0.00 | 0.11 | 0.07 |  | 0.00 | 0.24 | 0.15 |  | -0.01 | 0.11 | 0.07 |  | -0.06 | 0.24 | 0.15 |  | -0.04 | 0.11 | 0.07 |  | -0.19 | 0.25 | 0.17 |
| **(σ²,τ²) = (0.10;0.01)** | **Naïve Kaplan-Meier** | 0.00 | 0.08 | 0.08 |  | 0.01 | 0.18 | 0.17 |  | 0.00 | 0.08 | 0.08 |  | 0.00 | 0.18 | 0.17 |  | 0.00 | 0.09 | 0.08 |  | 0.00 | 0.18 | 0.17 |
|  | **Pooled Kaplan-Meier** | 0.00 | 0.08 | 0.08 |  | 0.00 | 0.20 | 0.19 |  | 0.00 | 0.08 | 0.08 |  | 0.01 | 0.20 | 0.19 |  | 0.00 | 0.09 | 0.09 |  | 0.03 | 0.19 | 0.19 |
|  | **Pooled Exponential** | 0.00 | 0.07 | 0.08 |  | 0.00 | 0.18 | 0.18 |  | 0.00 | 0.07 | 0.08 |  | 0.01 | 0.19 | 0.18 |  | 0.00 | 0.08 | 0.08 |  | 0.03 | 0.18 | 0.18 |
|  | **Peto-quintile** | 0.00 | 0.08 | 0.07 |  | 0.00 | 0.16 | 0.15 |  | -0.01 | 0.08 | 0.07 |  | -0.05 | 0.17 | 0.16 |  | -0.03 | 0.08 | 0.08 |  | -0.14 | 0.17 | 0.17 |
| **(σ²,τ²) = (0.10;0.10)** | **Naïve Kaplan-Meier** | 0.00 | 0.11 | 0.08 |  | 0.00 | 0.27 | 0.17 |  | 0.00 | 0.11 | 0.08 |  | 0.00 | 0.26 | 0.17 |  | 0.00 | 0.11 | 0.08 |  | 0.01 | 0.26 | 0.17 |
|  | **Pooled Kaplan-Meier** | 0.00 | 0.11 | 0.11 |  | 0.00 | 0.27 | 0.27 |  | 0.00 | 0.11 | 0.11 |  | 0.00 | 0.26 | 0.26 |  | 0.01 | 0.11 | 0.11 |  | 0.02 | 0.27 | 0.26 |
|  | **Pooled Exponential** | 0.00 | 0.10 | 0.10 |  | 0.00 | 0.27 | 0.26 |  | 0.00 | 0.10 | 0.10 |  | 0.00 | 0.26 | 0.26 |  | 0.00 | 0.11 | 0.10 |  | 0.03 | 0.26 | 0.25 |
|  | **Peto-quintile** | 0.00 | 0.11 | 0.07 |  | 0.00 | 0.24 | 0.16 |  | -0.01 | 0.10 | 0.07 |  | -0.06 | 0.23 | 0.16 |  | -0.03 | 0.11 | 0.08 |  | -0.13 | 0.26 | 0.17 |

**Table S2. Simulation results for comparisons of methods in estimating the difference in restricted mean survival time (rmstD). Scenario with J = 20 trials and n_J_ = 100 patients per trial and with proportional hazards.**

A random effects meta-analysis model was used for Pooled Kaplan-Meier, Pooled Exponential and Peto-quintile.

*β*: Size of treatment effect (=log(HR)); *σ²*: baseline hazard heterogeneity; *τ²*: treatment effect heterogeneity; ASE: average standard error; CI: Confidence interval; ESE: empirical standard error; rmstD: difference in restricted mean survival time; *t^*^*: time horizon

**Table S3. Simulation results for comparisons of methods in estimating the difference in restricted mean survival time (rmstD). Scenario with J = 5 trials and n_J_ = 200 patients per trial and with non-proportional hazards.**

| **Heterogeneity**  **scenario** | **Methods** | $\boldsymbol{\beta}$ **= 0** | | | | | | | | $\boldsymbol{\beta}$ **= -0.2** | | | | | | | | $\boldsymbol{\beta}$ **= -0.7** | | | | | | |
| --- | --- | --- | --- | --- | --- | --- | --- | --- | --- | --- | --- | --- | --- | --- | --- | --- | --- | --- | --- | --- | --- | --- | --- | --- |
|  |  | **t* = 5**  **True *rmstD* = 0** | | | | **t* = 10**  **True *rmstD* = 0** | | | | **t* = 5**  **True *rmstD* = -0.1** | | | | **t* = 10**  **True *rmstD* = 0.1** | | | | **t* = 5**  **True *rmstD* = -0.3** | | | | **t* = 10**  **True *rmstD* = 0.3** | | |
|  |  | Bias | ESE | ASE |  | Bias | ESE | ASE |  | Bias | ESE | ASE |  | Bias | ESE | ASE |  | Bias | ESE | ASE |  | Bias | ESE | ASE |
| **(σ²,τ²) = (0.01;0.01)** | **Naïve Kaplan-Meier** | 0.00 | 0.12 | 0.11 |  | 0.00 | 0.28 | 0.24 |  | 0.00 | 0.11 | 0.11 |  | 0.01 | 0.27 | 0.24 |  | 0.00 | 0.12 | 0.11 |  | 0.00 | 0.28 | 0.24 |
|  | **Pooled Kaplan-Meier** | 0.00 | 0.12 | 0.13 |  | 0.01 | 0.29 | 0.30 |  | 0.00 | 0.12 | 0.13 |  | -0.01 | 0.29 | 0.30 |  | 0.01 | 0.12 | 0.13 |  | -0.05 | 0.29 | 0.29 |
|  | **Pooled Exponential** | 0.00 | 0.10 | 0.11 |  | 0.00 | 0.27 | 0.28 |  | 0.12 | 0.10 | 0.11 |  | 0.03 | 0.27 | 0.28 |  | 0.40 | 0.12 | 0.12 |  | 0.07 | 0.30 | 0.30 |
|  | **Peto-quintile** | 0.00 | 0.11 | 0.10 |  | 0.00 | 0.24 | 0.21 |  | 0.01 | 0.11 | 0.10 |  | -0.05 | 0.24 | 0.21 |  | 0.02 | 0.11 | 0.10 |  | -0.19 | 0.24 | 0.21 |
| **(σ²,τ²) = (0.01;0.10)** | **Naïve Kaplan-Meier** | 0.00 | 0.19 | 0.11 |  | 0.02 | 0.50 | 0.24 |  | -0.01 | 0.19 | 0.11 |  | -0.02 | 0.48 | 0.24 |  | 0.00 | 0.19 | 0.11 |  | 0.00 | 0.48 | 0.24 |
|  | **Pooled Kaplan-Meier** | 0.01 | 0.19 | 0.18 |  | 0.02 | 0.50 | 0.46 |  | -0.01 | 0.19 | 0.18 |  | -0.03 | 0.49 | 0.46 |  | 0.00 | 0.19 | 0.18 |  | -0.05 | 0.48 | 0.46 |
|  | **Pooled Exponential** | 0.00 | 0.19 | 0.17 |  | 0.01 | 0.49 | 0.45 |  | 0.11 | 0.18 | 0.17 |  | 0.00 | 0.48 | 0.45 |  | 0.39 | 0.20 | 0.18 |  | 0.05 | 0.50 | 0.47 |
|  | **Peto-quintile** | 0.00 | 0.18 | 0.10 |  | 0.01 | 0.43 | 0.22 |  | 0.00 | 0.18 | 0.10 |  | -0.07 | 0.41 | 0.22 |  | 0.02 | 0.18 | 0.10 |  | -0.18 | 0.42 | 0.22 |
| **(σ²,τ²) = (0.10;0.01)** | **Naïve Kaplan-Meier** | 0.00 | 0.12 | 0.11 |  | 0.00 | 0.28 | 0.24 |  | 0.01 | 0.12 | 0.11 |  | 0.01 | 0.28 | 0.24 |  | 0.00 | 0.12 | 0.11 |  | -0.01 | 0.27 | 0.24 |
|  | **Pooled Kaplan-Meier** | 0.00 | 0.12 | 0.13 |  | 0.00 | 0.29 | 0.29 |  | 0.01 | 0.12 | 0.12 |  | -0.01 | 0.29 | 0.29 |  | 0.00 | 0.12 | 0.13 |  | -0.07 | 0.28 | 0.29 |
|  | **Pooled Exponential** | 0.00 | 0.10 | 0.11 |  | 0.00 | 0.27 | 0.28 |  | 0.12 | 0.10 | 0.11 |  | 0.03 | 0.27 | 0.27 |  | 0.38 | 0.12 | 0.12 |  | 0.04 | 0.30 | 0.31 |
|  | **Peto-quintile** | 0.00 | 0.11 | 0.10 |  | 0.00 | 0.24 | 0.21 |  | 0.01 | 0.12 | 0.10 |  | -0.05 | 0.24 | 0.22 |  | 0.00 | 0.11 | 0.10 |  | -0.21 | 0.25 | 0.22 |
| **(σ²,τ²) = (0.10;0.10)** | **Naïve Kaplan-Meier** | -0.01 | 0.19 | 0.11 |  | -0.01 | 0.46 | 0.24 |  | 0.00 | 0.18 | 0.11 |  | -0.01 | 0.46 | 0.24 |  | 0.00 | 0.20 | 0.11 |  | 0.01 | 0.49 | 0.24 |
|  | **Pooled Kaplan-Meier** | -0.01 | 0.19 | 0.18 |  | -0.02 | 0.47 | 0.45 |  | 0.00 | 0.18 | 0.18 |  | -0.03 | 0.46 | 0.45 |  | 0.00 | 0.20 | 0.18 |  | -0.05 | 0.48 | 0.45 |
|  | **Pooled Exponential** | -0.01 | 0.18 | 0.17 |  | -0.01 | 0.45 | 0.44 |  | 0.11 | 0.18 | 0.17 |  | 0.00 | 0.45 | 0.44 |  | 0.38 | 0.20 | 0.18 |  | 0.05 | 0.50 | 0.46 |
|  | **Peto-quintile** | -0.01 | 0.18 | 0.10 |  | -0.01 | 0.41 | 0.22 |  | 0.00 | 0.18 | 0.11 |  | -0.06 | 0.41 | 0.22 |  | 0.01 | 0.19 | 0.10 |  | -0.18 | 0.44 | 0.22 |

A random effects meta-analysis model was used for Pooled Kaplan-Meier, Pooled Exponential and Peto-quintile. Columns in grey (*β* = 0 and *β* = -0.7) are also shown in Table 1 in the main manuscript.

*β*: Size of treatment effect (=log(HR)); *σ²*: baseline hazard heterogeneity; *τ²*: treatment effect heterogeneity; ASE: average standard error; CI: Confidence interval; ESE: empirical standard error; rmstD: difference in restricted mean survival time; *t^*^*: time horizon

| **Heterogeneity**  **scenario** | **Methods** | $\boldsymbol{\beta}$ **= 0** | | | | | | | | $\boldsymbol{\beta}$ **= -0.2** | | | | | | | | $\boldsymbol{\beta}$ **= -0.7** | | | | | | |
| --- | --- | --- | --- | --- | --- | --- | --- | --- | --- | --- | --- | --- | --- | --- | --- | --- | --- | --- | --- | --- | --- | --- | --- | --- |
|  |  | **t* = 5**  **True *rmstD* = 0** | | | | **t* = 10**  **True *rmstD* = 0** | | | | **t* = 5**  **True *rmstD* = -0.1** | | | | **t* = 10**  **True *rmstD* = 0.1** | | | | **t* = 5**  **True *rmstD* = -0.3** | | | | **t* = 10**  **True *rmstD* = 0.3** | | |
|  |  | Bias | ESE | ASE |  | Bias | ESE | ASE |  | Bias | ESE | ASE |  | Bias | ESE | ASE |  | Bias | ESE | ASE |  | Bias | ESE | ASE |
| **(σ²,τ²) = (0.01;0.01)** | **Naïve Kaplan-Meier** | 0.00 | 0.08 | 0.08 |  | -0.01 | 0.18 | 0.17 |  | 0.00 | 0.08 | 0.08 |  | 0.01 | 0.19 | 0.17 |  | 0.00 | 0.08 | 0.08 |  | 0.01 | 0.18 | 0.17 |
|  | **Pooled Kaplan-Meier** | 0.00 | 0.08 | 0.08 |  | -0.01 | 0.20 | 0.20 |  | 0.00 | 0.08 | 0.08 |  | -0.01 | 0.20 | 0.20 |  | 0.01 | 0.08 | 0.09 |  | -0.05 | 0.20 | 0.20 |
|  | **Pooled Exponential** | 0.00 | 0.07 | 0.07 |  | -0.01 | 0.18 | 0.19 |  | 0.12 | 0.07 | 0.07 |  | 0.03 | 0.19 | 0.19 |  | 0.41 | 0.08 | 0.08 |  | 0.08 | 0.20 | 0.20 |
|  | **Peto-quintile** | 0.00 | 0.08 | 0.07 |  | -0.01 | 0.16 | 0.15 |  | 0.01 | 0.08 | 0.07 |  | -0.05 | 0.17 | 0.15 |  | 0.02 | 0.08 | 0.07 |  | -0.19 | 0.16 | 0.15 |
| **(σ²,τ²) = (0.01;0.10)** | **Naïve Kaplan-Meier** | 0.00 | 0.11 | 0.08 |  | 0.00 | 0.26 | 0.17 |  | 0.00 | 0.11 | 0.08 |  | 0.01 | 0.27 | 0.17 |  | 0.00 | 0.11 | 0.08 |  | 0.00 | 0.26 | 0.17 |
|  | **Pooled Kaplan-Meier** | 0.00 | 0.11 | 0.11 |  | 0.00 | 0.27 | 0.27 |  | 0.01 | 0.11 | 0.11 |  | -0.01 | 0.28 | 0.27 |  | 0.00 | 0.11 | 0.11 |  | -0.06 | 0.27 | 0.28 |
|  | **Pooled Exponential** | 0.00 | 0.10 | 0.10 |  | 0.00 | 0.26 | 0.27 |  | 0.12 | 0.10 | 0.10 |  | 0.03 | 0.27 | 0.27 |  | 0.40 | 0.11 | 0.11 |  | 0.06 | 0.28 | 0.28 |
|  | **Peto-quintile** | 0.00 | 0.10 | 0.07 |  | 0.00 | 0.23 | 0.15 |  | 0.01 | 0.10 | 0.07 |  | -0.04 | 0.24 | 0.15 |  | 0.01 | 0.10 | 0.07 |  | -0.18 | 0.23 | 0.15 |
| **(σ²,τ²) = (0.10;0.01)** | **Naïve Kaplan-Meier** | 0.00 | 0.08 | 0.08 |  | -0.01 | 0.18 | 0.17 |  | 0.00 | 0.08 | 0.08 |  | 0.00 | 0.19 | 0.17 |  | 0.00 | 0.08 | 0.08 |  | -0.01 | 0.18 | 0.17 |
|  | **Pooled Kaplan-Meier** | 0.00 | 0.08 | 0.08 |  | -0.01 | 0.20 | 0.19 |  | 0.00 | 0.08 | 0.08 |  | -0.02 | 0.19 | 0.19 |  | 0.00 | 0.08 | 0.08 |  | -0.07 | 0.20 | 0.19 |
|  | **Pooled Exponential** | 0.00 | 0.07 | 0.07 |  | -0.01 | 0.18 | 0.18 |  | 0.12 | 0.07 | 0.07 |  | 0.02 | 0.18 | 0.18 |  | 0.39 | 0.08 | 0.07 |  | 0.05 | 0.20 | 0.19 |
|  | **Peto-quintile** | 0.00 | 0.08 | 0.07 |  | -0.01 | 0.17 | 0.15 |  | 0.00 | 0.08 | 0.07 |  | -0.05 | 0.17 | 0.15 |  | 0.00 | 0.08 | 0.07 |  | -0.20 | 0.17 | 0.15 |
| **(σ²,τ²) = (0.10;0.10)** | **Naïve Kaplan-Meier** | 0.00 | 0.11 | 0.08 |  | -0.01 | 0.26 | 0.17 |  | 0.00 | 0.11 | 0.08 |  | 0.01 | 0.27 | 0.17 |  | 0.00 | 0.12 | 0.08 |  | -0.01 | 0.27 | 0.17 |
|  | **Pooled Kaplan-Meier** | 0.00 | 0.11 | 0.11 |  | -0.01 | 0.27 | 0.27 |  | 0.01 | 0.11 | 0.11 |  | 0.00 | 0.28 | 0.27 |  | 0.00 | 0.12 | 0.11 |  | -0.07 | 0.27 | 0.27 |
|  | **Pooled Exponential** | 0.00 | 0.10 | 0.10 |  | -0.01 | 0.26 | 0.26 |  | 0.12 | 0.10 | 0.10 |  | 0.03 | 0.27 | 0.26 |  | 0.38 | 0.11 | 0.11 |  | 0.04 | 0.28 | 0.27 |
|  | **Peto-quintile** | 0.00 | 0.10 | 0.07 |  | -0.01 | 0.24 | 0.16 |  | 0.01 | 0.11 | 0.07 |  | -0.04 | 0.24 | 0.16 |  | 0.00 | 0.11 | 0.07 |  | -0.20 | 0.25 | 0.16 |

**Table S4. Simulation results for comparisons of methods in estimating the difference in restricted mean survival time (rmstD). Scenario with J = 20 trials and n_J_ = 100 patients per trial and with non-proportional hazards.**

A random effects meta-analysis model was used for Pooled Kaplan-Meier, Pooled Exponential and Peto-quintile.

*β*: Size of treatment effect (=log(HR)); *σ²*: baseline hazard heterogeneity; *τ²*: treatment effect heterogeneity; ASE: average standard error; CI: Confidence interval; ESE: empirical standard error; rmstD: difference in restricted mean survival time; *t^*^*: time horizon

**Table S5. Simulation results for comparisons of methods in estimating the difference in restricted mean survival time.
Scenario with 5 trials and 200 patients per trial, with a deleterious treatment effect and with proportional hazards.**

| **Heterogeneity**  **scenario** | **Methods** | $\boldsymbol{\beta}$ **= 0.2** | | | | | | |  | $\boldsymbol{\beta}$ **= 0.7** | | | | | | |
| --- | --- | --- | --- | --- | --- | --- | --- | --- | --- | --- | --- | --- | --- | --- | --- | --- |
|  |  | ***t^*^* = 5**  **True *rmstD* = -0.2** | | |  | ***t^*^* = 10**  **True *rmstD* = -0.6** | | |  | ***t^*^* = 5**  **True *rmstD* = -0.8** | | |  | ***t^*^* = 10**  **True *rmstD* = -2.0** | | |
|  |  | Bias | ESE | ASE |  | Bias | ESE | ASE |  | Bias | ESE | ASE |  | Bias | ESE | ASE |
| **(σ²,τ²) = (0.01;0.01)** | **Naïve Kaplan-Meier** | 0.00 | 0.12 | 0.11 |  | 0.00 | 0.28 | 0.24 |  | 0.00 | 0.12 | 0.11 |  | 0.01 | 0.28 | 0.23 |
|  | **Pooled Kaplan-Meier** | 0.00 | 0.12 | 0.13 |  | -0.01 | 0.29 | 0.30 |  | 0.00 | 0.12 | 0.13 |  | 0.00 | 0.30 | 0.29 |
|  | **Pooled Exponential** | 0.00 | 0.10 | 0.11 |  | -0.01 | 0.27 | 0.28 |  | 0.00 | 0.11 | 0.11 |  | 0.00 | 0.28 | 0.28 |
|  | **Peto-quintile** | 0.02 | 0.11 | 0.10 |  | 0.08 | 0.24 | 0.21 |  | 0.04 | 0.12 | 0.10 |  | 0.22 | 0.27 | 0.23 |
| **(σ²,τ²) = (0.01;0.10)** | **Naïve Kaplan-Meier** | 0.00 | 0.19 | 0.11 |  | 0.01 | 0.49 | 0.24 |  | 0.00 | 0.19 | 0.11 |  | 0.00 | 0.48 | 0.23 |
|  | **Pooled Kaplan-Meier** | 0.00 | 0.20 | 0.18 |  | 0.00 | 0.50 | 0.46 |  | 0.00 | 0.20 | 0.18 |  | 0.00 | 0.48 | 0.45 |
|  | **Pooled Exponential** | 0.00 | 0.19 | 0.17 |  | 0.00 | 0.48 | 0.45 |  | 0.00 | 0.19 | 0.17 |  | -0.01 | 0.47 | 0.44 |
|  | **Peto-quintile** | 0.01 | 0.18 | 0.10 |  | 0.07 | 0.43 | 0.22 |  | 0.04 | 0.19 | 0.11 |  | 0.20 | 0.46 | 0.23 |
| **(σ²,τ²) = (0.10;0.01)** | **Naïve Kaplan-Meier** | -0.01 | 0.12 | 0.11 |  | -0.01 | 0.27 | 0.24 |  | 0.00 | 0.13 | 0.11 |  | 0.00 | 0.27 | 0.23 |
|  | **Pooled Kaplan-Meier** | 0.00 | 0.12 | 0.13 |  | -0.01 | 0.28 | 0.29 |  | 0.00 | 0.13 | 0.13 |  | 0.00 | 0.29 | 0.29 |
|  | **Pooled Exponential** | 0.00 | 0.10 | 0.11 |  | -0.01 | 0.26 | 0.28 |  | 0.01 | 0.12 | 0.12 |  | -0.01 | 0.26 | 0.27 |
|  | **Peto-quintile** | 0.01 | 0.11 | 0.10 |  | 0.05 | 0.23 | 0.22 |  | 0.03 | 0.13 | 0.11 |  | 0.16 | 0.26 | 0.23 |
| **(σ²,τ²) = (0.10;0.10)** | **Naïve Kaplan-Meier** | 0.00 | 0.18 | 0.11 |  | -0.01 | 0.44 | 0.24 |  | 0.00 | 0.20 | 0.11 |  | 0.00 | 0.46 | 0.24 |
|  | **Pooled Kaplan-Meier** | 0.00 | 0.18 | 0.18 |  | -0.01 | 0.45 | 0.45 |  | 0.00 | 0.20 | 0.19 |  | 0.00 | 0.45 | 0.44 |
|  | **Pooled Exponential** | 0.00 | 0.17 | 0.17 |  | 0.00 | 0.44 | 0.44 |  | 0.00 | 0.18 | 0.18 |  | -0.01 | 0.44 | 0.43 |
|  | **Peto-quintile** | 0.01 | 0.17 | 0.10 |  | 0.06 | 0.40 | 0.22 |  | 0.03 | 0.19 | 0.11 |  | 0.14 | 0.45 | 0.24 |

A random effects meta-analysis model was used for Pooled Kaplan-Meier, Pooled Exponential and Peto-quintile.

*β*: Size of treatment effect (=log(HR)); *σ²*: baseline hazard heterogeneity; *τ²*: treatment effect heterogeneity; ASE: average standard error; CI: Confidence interval; ESE: empirical standard error; rmstD: difference in restricted mean survival time; *t^*^*: time horizon

**Table S6. Simulation results for comparisons of methods in estimating the difference in restricted mean survival time (rmstD). Scenario with J = 5 trials and n_J_ = 200 patients per trial, with proportional hazards and with correlation between the two random effects.**

| **Heterogeneity**  **scenario** | **Methods** | $\boldsymbol{\beta}$ **= 0** | | | | | | | | $\boldsymbol{\beta}$ **= -0.2** | | | | | | | | $\boldsymbol{\beta}$ **= -0.7** | | | | | | |
| --- | --- | --- | --- | --- | --- | --- | --- | --- | --- | --- | --- | --- | --- | --- | --- | --- | --- | --- | --- | --- | --- | --- | --- | --- |
|  |  | **t* = 5**  **True *rmstD* = 0** | | | | **t* = 10**  **True *rmstD* = 0** | | | | **t* = 5**  **True *rmstD* = 0.2** | | | | **t* = 10**  **True *rmstD* = 0.6** | | | | **t* = 5**  **True *rmstD* = 0.8** | | | | **t* = 10**  **True *rmstD* = 2.0** | | |
|  |  | Bias | ESE | ASE |  | Bias | ESE | ASE |  | Bias | ESE | ASE |  | Bias | ESE | ASE |  | Bias | ESE | ASE |  | Bias | ESE | ASE |
| **(σ²,τ²) = (0.01;0.01)** | **Naïve Kaplan-Meier** | 0.00 | 0.12 | 0.11 |  | 0.00 | 0.28 | 0.24 |  | 0.00 | 0.12 | 0.11 |  | 0.00 | 0.29 | 0.24 |  | 0.01 | 0.13 | 0.11 |  | 0.01 | 0.27 | 0.23 |
|  | **Pooled Kaplan-Meier** | 0.00 | 0.12 | 0.13 |  | 0.00 | 0.29 | 0.30 |  | 0.00 | 0.13 | 0.13 |  | 0.00 | 0.30 | 0.30 |  | 0.01 | 0.13 | 0.13 |  | 0.02 | 0.30 | 0.29 |
|  | **Pooled Exponential** | -0.01 | 0.10 | 0.11 |  | 0.00 | 0.27 | 0.28 |  | 0.00 | 0.11 | 0.11 |  | 0.00 | 0.28 | 0.28 |  | 0.01 | 0.11 | 0.11 |  | 0.03 | 0.27 | 0.28 |
|  | **Peto-quintile** | 0.00 | 0.11 | 0.10 |  | 0.00 | 0.24 | 0.21 |  | -0.01 | 0.12 | 0.10 |  | -0.07 | 0.25 | 0.21 |  | -0.04 | 0.12 | 0.11 |  | -0.20 | 0.26 | 0.23 |
| **(σ²,τ²) = (0.01;0.10)** | **Naïve Kaplan-Meier** | 0.00 | 0.19 | 0.11 |  | 0.02 | 0.47 | 0.24 |  | 0.00 | 0.19 | 0.11 |  | 0.00 | 0.46 | 0.24 |  | 0.01 | 0.19 | 0.11 |  | 0.02 | 0.46 | 0.23 |
|  | **Pooled Kaplan-Meier** | 0.00 | 0.19 | 0.18 |  | 0.02 | 0.47 | 0.45 |  | -0.01 | 0.19 | 0.19 |  | 0.00 | 0.48 | 0.46 |  | 0.01 | 0.19 | 0.19 |  | 0.03 | 0.47 | 0.45 |
|  | **Pooled Exponential** | 0.00 | 0.18 | 0.17 |  | 0.02 | 0.46 | 0.44 |  | 0.00 | 0.18 | 0.17 |  | 0.00 | 0.46 | 0.45 |  | 0.01 | 0.19 | 0.18 |  | 0.03 | 0.46 | 0.43 |
|  | **Peto-quintile** | 0.00 | 0.18 | 0.10 |  | 0.02 | 0.41 | 0.22 |  | -0.02 | 0.18 | 0.10 |  | -0.07 | 0.41 | 0.22 |  | -0.03 | 0.19 | 0.11 |  | -0.16 | 0.44 | 0.24 |
| **(σ²,τ²) = (0.10;0.01)** | **Naïve Kaplan-Meier** | 0.00 | 0.12 | 0.11 |  | -0.01 | 0.27 | 0.24 |  | 0.00 | 0.13 | 0.11 |  | 0.01 | 0.28 | 0.24 |  | 0.01 | 0.15 | 0.11 |  | 0.01 | 0.29 | 0.23 |
|  | **Pooled Kaplan-Meier** | -0.01 | 0.12 | 0.13 |  | 0.01 | 0.28 | 0.29 |  | 0.00 | 0.13 | 0.13 |  | 0.02 | 0.30 | 0.30 |  | 0.01 | 0.15 | 0.15 |  | 0.03 | 0.30 | 0.29 |
|  | **Pooled Exponential** | -0.01 | 0.10 | 0.11 |  | 0.00 | 0.27 | 0.28 |  | 0.00 | 0.11 | 0.11 |  | 0.02 | 0.27 | 0.28 |  | 0.00 | 0.14 | 0.13 |  | 0.03 | 0.28 | 0.28 |
|  | **Peto-quintile** | 0.00 | 0.11 | 0.10 |  | 0.01 | 0.24 | 0.22 |  | -0.01 | 0.12 | 0.10 |  | -0.04 | 0.25 | 0.22 |  | -0.02 | 0.15 | 0.11 |  | -0.12 | 0.28 | 0.23 |
| **(σ²,τ²) = (0.10;0.10)** | **Naïve Kaplan-Meier** | 0.00 | 0.19 | 0.11 |  | 0.02 | 0.46 | 0.24 |  | -0.02 | 0.21 | 0.11 |  | -0.03 | 0.49 | 0.24 |  | 0.02 | 0.24 | 0.11 |  | 0.04 | 0.50 | 0.24 |
|  | **Pooled Kaplan-Meier** | 0.00 | 0.19 | 0.19 |  | 0.01 | 0.47 | 0.46 |  | -0.02 | 0.21 | 0.20 |  | -0.03 | 0.49 | 0.46 |  | 0.01 | 0.24 | 0.23 |  | 0.04 | 0.50 | 0.47 |
|  | **Pooled Exponential** | 0.00 | 0.18 | 0.18 |  | 0.02 | 0.46 | 0.45 |  | -0.02 | 0.20 | 0.19 |  | -0.03 | 0.49 | 0.45 |  | 0.01 | 0.24 | 0.22 |  | 0.04 | 0.50 | 0.46 |
|  | **Peto-quintile** | 0.00 | 0.18 | 0.11 |  | 0.04 | 0.42 | 0.22 |  | -0.03 | 0.20 | 0.11 |  | -0.06 | 0.45 | 0.23 |  | -0.01 | 0.25 | 0.11 |  | -0.04 | 0.52 | 0.25 |

A random effects meta-analysis model was used for Pooled Kaplan-Meier, Pooled Exponential and Peto-quintile.

*β*: Size of treatment effect (=log(HR)); *σ²*: baseline hazard heterogeneity; *τ²*: treatment effect heterogeneity; ASE: average standard error; CI: Confidence interval; ESE: empirical standard error; rmstD: difference in restricted mean survival time; *t^*^*: time horizon

**Table S7. Simulation results for comparisons of methods in estimating the difference in restricted mean survival time (rmstD). Scenario with J = 5 trials and n_J_ = 200 patients per trial and with proportional hazards.**

| **Heterogeneity**  **scenario** | **Methods** | $\boldsymbol{\beta}$ **= 0** | | | | | | | | $\boldsymbol{\beta}$ **= -0.2** | | | | | | | | $\boldsymbol{\beta}$ **= -0.7** | | | | | | |
| --- | --- | --- | --- | --- | --- | --- | --- | --- | --- | --- | --- | --- | --- | --- | --- | --- | --- | --- | --- | --- | --- | --- | --- | --- |
|  |  | **t* = 5**  **True *rmstD* = 0** | | | | **t* = 10**  **True *rmstD* = 0** | | | | **t* = 5**  **True *rmstD* = 0.2** | | | | **t* = 10**  **True *rmstD* = 0.6** | | | | **t* = 5**  **True *rmstD* = 0.8** | | | | **t* = 10**  **True *rmstD* = 2.0** | | |
|  |  | Bias | ESE | ASE |  | Bias | ESE | ASE |  | Bias | ESE | ASE |  | Bias | ESE | ASE |  | Bias | ESE | ASE |  | Bias | ESE | ASE |
| **(σ²,τ²) = (0.01;0.01)** | **Naïve Kaplan-Meier** | 0.00 | 0.12 | 0.11 |  | 0.00 | 0.28 | 0.24 |  | 0.00 | 0.12 | 0.11 |  | 0.00 | 0.29 | 0.24 |  | 0.01 | 0.12 | 0.11 |  | 0.01 | 0.28 | 0.23 |
|  | **Pooled Kaplan-Meier** | 0.00 | 0.12 | 0.11 |  | 0.00 | 0.29 | 0.23 |  | 0.00 | 0.12 | 0.11 |  | 0.01 | 0.31 | 0.23 |  | 0.01 | 0.12 | 0.11 |  | 0.03 | 0.30 | 0.23 |
|  | **Pooled Exponential** | 0.00 | 0.10 | 0.09 |  | 0.00 | 0.27 | 0.24 |  | 0.00 | 0.11 | 0.09 |  | 0.01 | 0.28 | 0.24 |  | 0.01 | 0.11 | 0.09 |  | 0.03 | 0.27 | 0.23 |
|  | **Peto-quintile** | 0.00 | 0.11 | 0.10 |  | 0.00 | 0.24 | 0.21 |  | -0.01 | 0.12 | 0.10 |  | -0.07 | 0.25 | 0.21 |  | -0.04 | 0.12 | 0.10 |  | -0.21 | 0.26 | 0.22 |
| **(σ²,τ²) = (0.01;0.10)** | **Naïve Kaplan-Meier** | 0.00 | 0.19 | 0.11 |  | 0.01 | 0.49 | 0.24 |  | 0.00 | 0.19 | 0.11 |  | 0.01 | 0.48 | 0.24 |  | 0.00 | 0.19 | 0.11 |  | 0.00 | 0.48 | 0.23 |
|  | **Pooled Kaplan-Meier** | 0.00 | 0.20 | 0.11 |  | 0.01 | 0.51 | 0.23 |  | 0.00 | 0.19 | 0.11 |  | 0.02 | 0.50 | 0.23 |  | 0.01 | 0.20 | 0.11 |  | 0.06 | 0.49 | 0.23 |
|  | **Pooled Exponential** | 0.00 | 0.19 | 0.09 |  | 0.01 | 0.50 | 0.24 |  | 0.00 | 0.18 | 0.09 |  | 0.02 | 0.49 | 0.23 |  | 0.00 | 0.19 | 0.09 |  | 0.05 | 0.49 | 0.23 |
|  | **Peto-quintile** | 0.00 | 0.18 | 0.10 |  | 0.00 | 0.42 | 0.21 |  | -0.01 | 0.18 | 0.10 |  | -0.07 | 0.42 | 0.21 |  | -0.04 | 0.19 | 0.10 |  | -0.21 | 0.45 | 0.22 |
| **(σ²,τ²) = (0.10;0.01)** | **Naïve Kaplan-Meier** | -0.01 | 0.12 | 0.11 |  | -0.01 | 0.28 | 0.24 |  | 0.00 | 0.12 | 0.11 |  | 0.01 | 0.27 | 0.24 |  | 0.00 | 0.14 | 0.11 |  | 0.01 | 0.28 | 0.24 |
|  | **Pooled Kaplan-Meier** | -0.01 | 0.12 | 0.11 |  | -0.01 | 0.28 | 0.23 |  | 0.00 | 0.12 | 0.11 |  | 0.00 | 0.29 | 0.23 |  | 0.00 | 0.14 | 0.11 |  | 0.02 | 0.29 | 0.22 |
|  | **Pooled Exponential** | 0.00 | 0.10 | 0.09 |  | -0.01 | 0.26 | 0.23 |  | 0.00 | 0.10 | 0.09 |  | 0.01 | 0.26 | 0.23 |  | -0.01 | 0.12 | 0.09 |  | 0.02 | 0.27 | 0.22 |
|  | **Peto-quintile** | -0.01 | 0.11 | 0.10 |  | -0.01 | 0.23 | 0.21 |  | -0.01 | 0.12 | 0.10 |  | -0.06 | 0.24 | 0.21 |  | -0.03 | 0.13 | 0.10 |  | -0.15 | 0.26 | 0.23 |
| **(σ²,τ²) = (0.10;0.10)** | **Naïve Kaplan-Meier** | 0.00 | 0.18 | 0.11 |  | 0.00 | 0.45 | 0.24 |  | -0.01 | 0.19 | 0.11 |  | 0.00 | 0.48 | 0.24 |  | 0.01 | 0.19 | 0.11 |  | 0.01 | 0.44 | 0.24 |
|  | **Pooled Kaplan-Meier** | 0.00 | 0.18 | 0.11 |  | 0.00 | 0.46 | 0.23 |  | -0.01 | 0.19 | 0.11 |  | 0.01 | 0.49 | 0.23 |  | 0.01 | 0.20 | 0.10 |  | 0.05 | 0.45 | 0.22 |
|  | **Pooled Exponential** | 0.00 | 0.17 | 0.09 |  | 0.00 | 0.45 | 0.23 |  | 0.00 | 0.18 | 0.09 |  | 0.01 | 0.48 | 0.23 |  | 0.00 | 0.19 | 0.09 |  | 0.05 | 0.45 | 0.22 |
|  | **Peto-quintile** | 0.00 | 0.17 | 0.10 |  | 0.00 | 0.40 | 0.21 |  | -0.02 | 0.18 | 0.10 |  | -0.06 | 0.42 | 0.21 |  | -0.02 | 0.19 | 0.11 |  | -0.15 | 0.43 | 0.23 |

A fixed effect meta-analysis model was used for Pooled Kaplan-Meier, Pooled Exponential and Peto-quintile.

*β*: Size of treatment effect (=log(HR)); *σ²*: baseline hazard heterogeneity; *τ²*: treatment effect heterogeneity; ASE: average standard error; CI: Confidence interval; ESE: empirical standard error; rmstD: difference in restricted mean survival time difference in restricted mean survival time; *t^*^*: time horizon

**
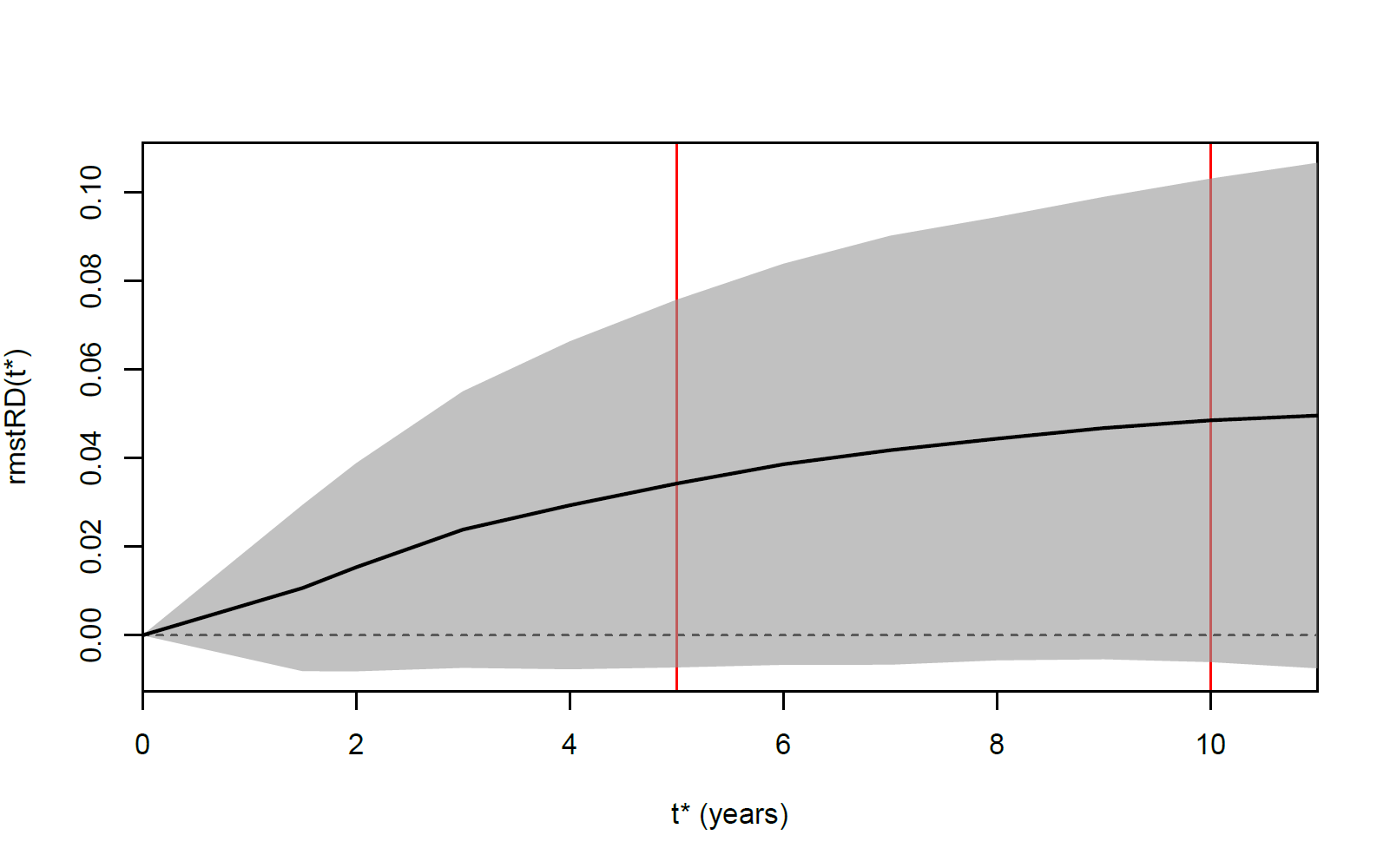
Figure S1. Relative difference in restricted mean survival time estimated using the Pooled Kaplan-Meier method with random effects as a function of the time horizon *t^*^* in the MAC-NPC meta-analysis.**

The solid black line represents the *rmstRD*(*t^*^*) plotted as a function of the horizon *t^*^*. The dashed horizontal line indicates the absence of a treatment effect (*rmstRD*(*t^*^*)=0). The grey area corresponds to the pointwise 95% confidence interval.

MAC-NPC: Meta-Analysis of Chemotherapy in Nasopharynx Carcinoma; rmstRD: relative difference in restricted mean survival time.

**
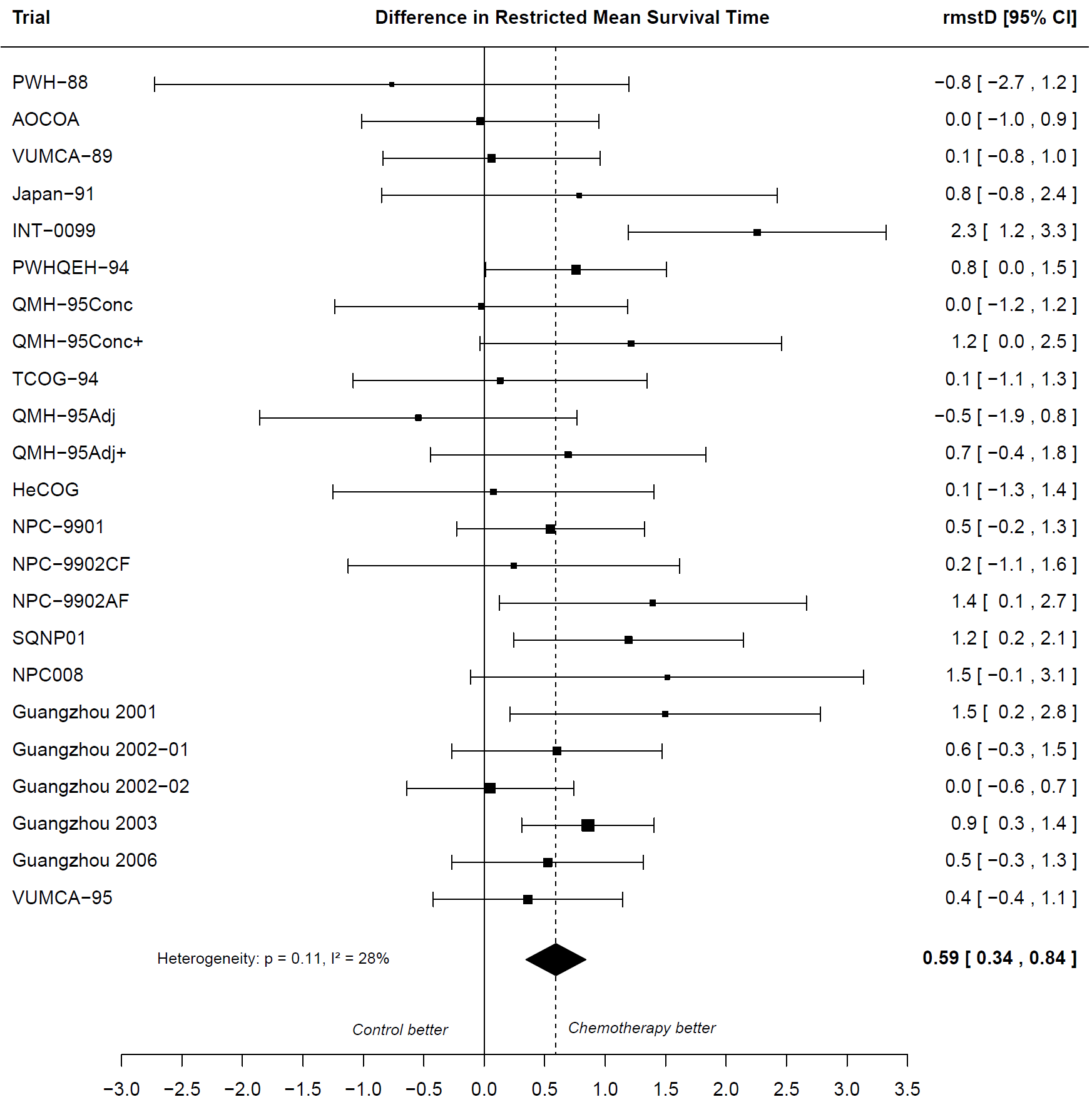
**

**Figure S2. Forest plot for differences in restricted mean survival time estimated at 10 years using the Pooled Kaplan-Meier method with random effect applied to the MAC-NPC2 meta-analysis.**

Each trial is represented by a square, the center of which denotes the difference in restricted mean survival time (rmstD) for that trial comparison, with the horizontal lines showing the 95% confidence intervals (CI). The size of the square is directly proportional to the amount of information contributed by the trial. The diamond represents the overall rmstD, with the center denoting the rmstD and the extremities the 95% CI. The rmstDs are expressed in years.
